# Independent and Combined Associations of Sleep Duration, Bedtime, and Polygenic Risk Score with the Risk of Hearing Loss among Middle-Aged and Old Chinese: The Dongfeng–Tongji Cohort Study

**DOI:** 10.34133/research.0178

**Published:** 2023-06-27

**Authors:** Miao Liu, Haiqing Zhang, Zhichao Wang, Tingting Mo, Xuefeng Lai, Yaling He, Minghui Jiang, Meian He, Weijia Kong, Tangchun Wu, Xiaomin Zhang

**Affiliations:** ^1^ Department of Occupational and Environmental Health, Ministry of Education Key Laboratory of Environment and Health, and State Key Laboratory of Environmental Health (Incubating), School of Public Health, Tongji Medical College, Huazhong University of Science and Technology, Wuhan, Hubei, China.; ^2^ Department of Otorhinolaryngology, Union Hospital, Tongji Medical College, Huazhong University of Science and Technology, Wuhan, Hubei, China.; ^3^ Department of Epidemiology and Biostatistics, School of Public Health, Tongji Medical College, Huazhong University of Science and Technology, Wuhan, Hubei, China.

## Abstract

Evidence available on the independent and combined associations of sleep duration, bedtime, and genetic predisposition with hearing loss was lacking. The present study included 15,827 participants from the Dongfeng–Tongji cohort study. Genetic risk was characterized by polygenic risk score (PRS) based on 37 genetic loci related to hearing loss. We conducted multivariate logistic regression models to assess the odds ratio (OR) for hearing loss with sleep duration and bedtime, as well as the joint association and interaction with PRS. Results showed that hearing loss was independently associated with sleeping ≥9 h/night compared to the recommended 7 to <8 h/night, and with bedtime ≤9:00 p.m. and >9:00 p.m. to 10:00 p.m. compared to those with bedtime >10:00 p.m. to 11:00 p.m., with estimated ORs of 1.25, 1.27, and 1.16, respectively. Meanwhile, the risk of hearing loss increased by 29% for each 5-risk allele increment of PRS. More importantly, joint analyses showed that the risk of hearing loss was 2-fold in sleep duration ≥9 h/night and high PRS, and 2.18-fold in bedtime ≤9:00 p.m. and high PRS. With significant joint effects of sleep duration and bedtime on hearing loss, we found an interaction of sleep duration with PRS in those with early bedtime and an interaction of bedtime with PRS in those with long sleep duration on hearing loss (*P*_int_ <0.05), and such relationships were more evident in high PRS. Similarly, the above relationships were also observed for age-related hearing loss and noise-induced hearing loss, particularly the latter. In addition, age-modified effects of sleep patterns on hearing loss were likewise observed, with stronger estimation among those aged <65 years. Accordingly, longer sleep duration, early bedtime, and high PRS were independently and jointly related to increased risk of hearing loss, suggesting the importance of considering both genetics and sleep pattern for risk assessment of hearing loss.

## Introduction

Hearing loss as the third largest cause of years lived with disability globally has affected 1.57 billion people by 2019, and is estimated to grow to 2.45 billion by 2050 [1]. The etiology of hearing loss is various and complex, including aging, noise, lifestyles, and heredity, and others [2,3]. The common modifiable risk factors, such as smoking, lack of regular physical exercise, and unhealthy diet, are related to a higher risk of hearing loss [4,5], whereas the influence of sleep on hearing loss is little known.

It is generally recommended that a sleep duration of 7 to 8 h/night and a bedtime of 10:00 p.m. to 11:00 p.m. would be the best for adults, while shorter or longer sleep duration and earlier or later bedtime might be harmful to human health [6,7]. However, only 3 studies have explored the relationship between sleep and hearing loss, which failed to achieve agreement [8–10]. A cross-sectional study presented that the association of longer sleep duration and high-frequency hearing loss was positive in American adults while it was the opposite in Chinese adults [9]. Another Japanese study showed that participants with longer sleep duration had a higher prevalence of hearing loss, but they only considered audiometric thresholds at a single frequency, such as 1 kHz or 4 kHz [10], while the cross-sectional study did not observe any significant results in 632 older American adults [8]. Notably, bedtime-affected biological rhythms also play a significant role in the development of diseases [7], but it is unknown whether different bedtimes have adverse effects on hearing loss.

Although environmental risk factors contribute the most to hearing loss risk, it is reported that heritability of up to 36% variation could account for hearing loss [11]. Hence, it is necessary to take genetic risk into account when exploring the effect of sleep duration and bedtime on hearing loss, which has not been covered yet. Recently, a large-scale genome-wide association study (GWAS) meta-analysis has identified 48 risk loci related to hearing loss [12], but each single nucleotide polymorphism (SNP) only has a very small effect. Polygenic risk score (PRS), combining many genetic variants, may have a significant impact on the risk of hearing loss [13]. Also, prior GWASs with hearing loss-related genetic risk were mostly reported in Europeans and Americans [12], which usually made interpretation of the results difficult for Asians.

As aging and noise are the most important risk factors of hearing loss, it is also necessary to separately evaluate the impact of sleep duration and bedtime on age-related hearing loss (ARHL) and noise-induced hearing loss (NIHL), which have different pathological processes [14,15]. The Dongfeng–Tongji (DFTJ) cohort study focused on the health of middle-aged and older people in one of the 3 largest automobile manufacturers in China [16], and collected detailed information on noise exposure through workplace monitoring. Therefore, based on the DFTJ cohort study, we proposed to investigate the independent and combined associations of sleep duration and bedtime with hearing loss. Besides, we generated hearing loss PRSs based on GWASs and explore the potential joint association and interaction of calculated PRS with sleep duration and bedtime on hearing loss.

## Results

### Characteristics of study participants

We listed the characteristics of the study subjects in Table 1. Overall, the age was 64.5 ± 8.3 years and 42.8% were male. The average body mass index (BMI) was 24.2 kg/m^2^; 12.3%, 6.0%, and 23.7% of the total subjects were current smokers, current drinkers, and taking ototoxic drugs, respectively. Those with hearing loss in the overall population, the ARHL subgroup, and the NIHL subgroup accounted for 47.8%, 46.1%, and 49.5%, respectively. Sleep duration of at least 9 h/night accounted for 26.5% and those who went to sleep before 9:00 p.m. or 10:00 p.m. accounted for 8.6% and 35.6%, respectively. Compared to those in the NIHL subgroup, participants in the ARHL subgroup were more likely to be older, be female, have a higher education level, have never smoked, have never drank, have an earlier bedtime, or have hypertension or hyperlipidemia. There was no significant difference in sleep duration between the 2 subgroups. We also present the baseline characteristics of sleep duration, bedtime, and PRS between hearing loss and normal hearing in Table S2. Results showed that the percentages of sleeping ≥9 h/night, bedtime before 10:00 p.m., and PRS were higher in those with hearing loss than in those with normal hearing (*P* < 0.001).

**Table 1.** Characteristics of study participants. Data were presented as means ± SD for normally distributed variables and numbers (percentages) for categorical variables.

Variables	Overall	ARHL subgroup	NIHL subgroup	*P*
Sample size	15,827	9,456	6,170	-
Age, years	64.5 ± 8.3	65.1 ± 8.2	63.5 ± 8.3	<0.001
Male, *n* (%)	6,769 (42.8)	3,583 (37.9)	3,088 (50.0)	<0.001
BMI, kg/m^2^	24.2 ± 3.3	24.3 ± 3.3	24.2 ± 3.3	<0.001
Education level, *n* (%)				<0.001
Primary school or below	3,333 (21.1)	1,972 (20.9)	1,307 (21.2)	
Middle school	5,710 (36.1)	3,018 (31.9)	2,627 (42.6)	
High school or beyond	6,784 (42.9)	4,466 (47.2)	2,236 (36.2)	
Smoking status, *n* (%)				<0.001
Current smoker	1,953 (12.3)	1,072 (11.3)	861 (14.0)	
Former smoker	2,287 (14.5)	1,109 (11.7)	1,143 (18.5)	
Never smoker	11,587 (73.2)	7,275 (76.9)	4,166 (67.5)	
Drinking status, *n* (%)				<0.001
Current drinker	956 (6.0)	527 (5.6)	413 (6.7)	
Former drinker	3,796 (24.0)	2,026 (21.4)	1,717 (27.8)	
Never drinker	11,075 (70.0)	6,903 (73.0)	4,040 (65.5)	
Regular exercise, *n* (%)	14,164 (89.5)	8,499 (89.9)	5,493 (89.0)	0.089
Take ototoxic drug, *n* (%)	3,749 (23.7)	2,217 (23.5)	1,472 (23.9)	0.553
Sleep duration, h/night, *n* (%)				0.202
<7	910 (5.8)	527 (5.6)	370 (6.0)	
7 to <8	3,949 (25.0)	2,398 (25.4)	1,504 (24.4)	
8 to <9	6,759 (42.7)	4,063 (43.0)	2,617 (42.4)	
≥9	4,209 (26.5)	2,468 (26.0)	1,679 (27.2)	
Bedtime, p.m., *n* (%)				0.009
≤9:00	1,363 (8.6)	819 (8.7)	525 (8.5)	
>9:00 to 10:00	5,635 (35.6)	3,382 (35.8)	2,169 (35.2)	
>10:00 to 11:00	6,780 (42.8)	4,100 (43.4)	2,607 (42.3)	
>11:00	2,049 (12.9)	1,155 (12.2)	869 (14.1)	
Sleep quality, *n* (%)				0.479
Good	5,286 (33.4)	3,179 (33.6)	2,050 (33.2)	
Fair	8,168 (51.6)	4,889 (51.7)	3,171 (51.4)	
Poor	2,373 (15.0)	1,388 (14.7)	949 (15.4)	
Hearing loss, *n* (%)	7,559 (47.8)	4,355 (46.1)	3,056 (49.5)	<0.001
Hypertension, *n* (%)	10,240 (64.7)	6,175 (65.3)	3,920 (63.5)	0.024
Type 2 diabetes, *n* (%)	3,430 (21.7)	2,048 (21.7)	1,330 (21.6)	0.879
Hyperlipidemia, *n* (%)	8,462 (53.5)	5,149 (54.5)	3,203 (51.9)	0.002
PRS	34.4 ± 4.4	34.4 ± 4.4	34.4 ± 4.4	0.619

ARHL, age-related hearing loss; NIHL, noise-induced hearing loss; BMI, body mass index; PRS, polygenic risk score; SD, standard deviation; IQR, interquartile range.

### Associations of sleep duration and bedtime with hearing loss

**Fig. 1.**
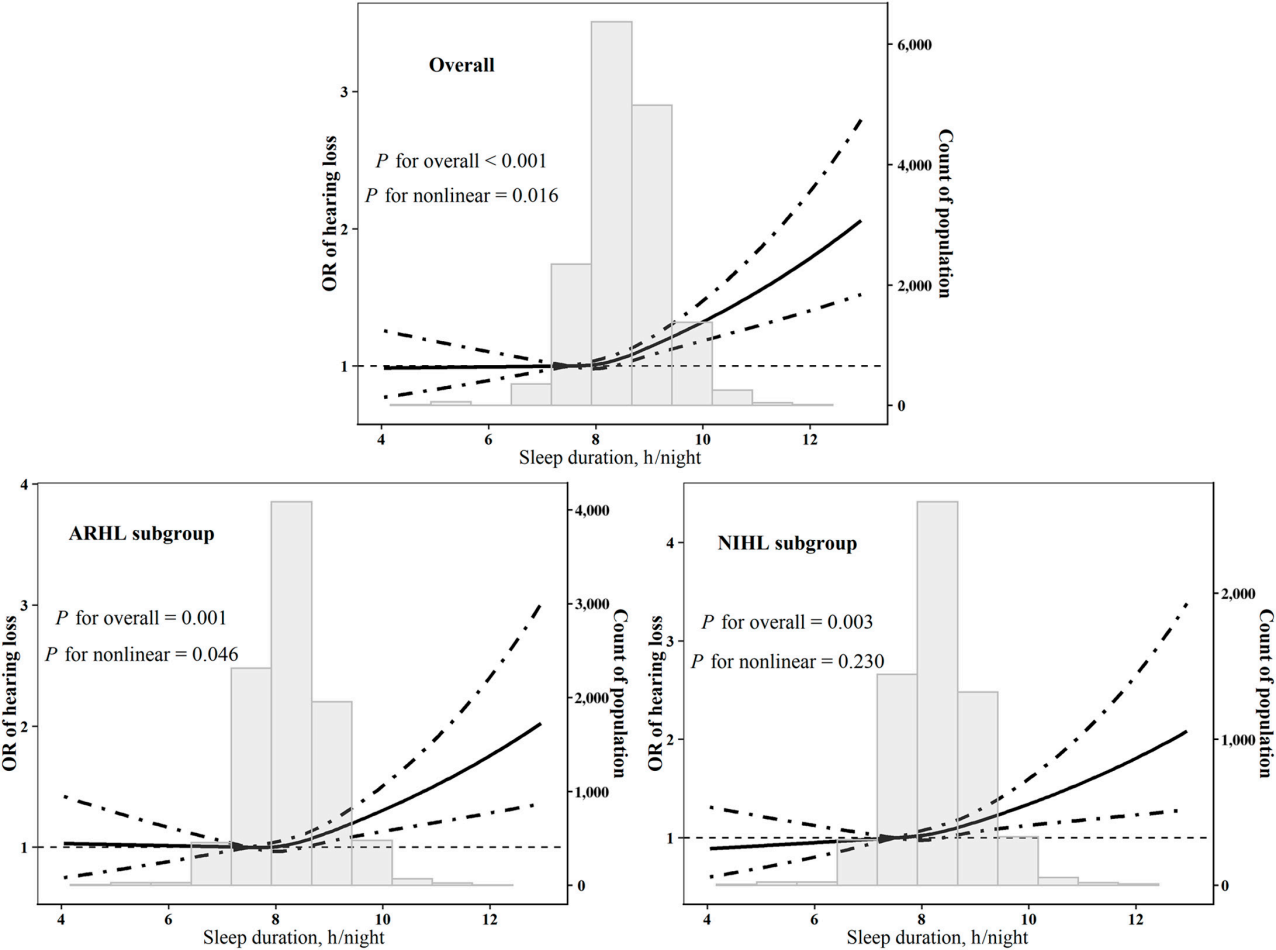
Restricted cubic spline for associations of sleep duration with hearing loss. Model adjusted for age, gender, body mass index, education level, smoking status, drinking status, regular exercise, sleep quality, hypertension, diabetes, hyperlipidemia, taking ototoxic drug, and occupational noise exposure (not in ARHL/NIHL subgroup analyses). Knots were placed at the 25th, 50th, and 75th percentiles of sleep duration distribution, and the reference was set at 7.5 h/night.

As shown in Table 2, after adjusting for multiple covariates, compared with sleeping 7 to <8 h/night, the odds ratio (OR) (95% CIs) of hearing loss for sleeping ≥9 h/night was the strongest with a significant estimation of 1.25 (1.14, 1.38) among the overall population. We also observed that the ORs (95% CIs) of hearing loss were 1.22 (1.08, 1.38) in the ARHL subgroup and 1.30 (1.11, 1.52) in the NIHL subgroup when comparing sleeping ≥9 h/night with 7 to <8 h/night. Meanwhile, among the overall population, compared to those with bedtime >10:00 p.m. to 11:00 p.m., the strongest association showed significance between bedtime ≤9:00 p.m. and risk of hearing loss with an OR (95% CIs) of 1.27 (1.11, 1.45). Bedtime >9:00 p.m. to 10:00 p.m. was related to the risk of hearing loss with an OR (95% CIs) of 1.16 (1.08, 1.26) when compared to the bedtime >10:00 p.m. to 11:00 p.m. in the overall population. Similar results were also found in the ARHL and NIHL subgroups. Compared with bedtime >10:00 p.m. to 11:00 p.m., the ORs (95% CIs) of hearing loss were 1.32 (1.11, 1.56) for ≤9:00 p.m. and 1.15 (1.04, 1.27) for >9:00 p.m. to 10:00 p.m. in the ARHL subgroup and 1.18 (1.04, 1.34) for >9:00 p.m. to 10:00 p.m. in the NIHL subgroup. Also, we found the linear association of sleep duration and hearing loss in the NIHL subgroup (*P* for overall = 0.003, *P* for nonlinearity = 0.230), while the dose–response relationships of sleep duration and hearing loss in the overall population and the ARHL subgroup were nonlinear (*P* for overall ≤ 0.001, *P* for nonlinearity = 0.016 and 0.046, respectively), with a sharp increase when sleep duration is around 9 h/night (Fig. 1).

**Table 2.** Associations of sleep duration and bedtime with hearing loss.

Variables	Overall (*n* = 15,827)	ARHL subgroup (*n* = 9,456)	NIHL subgroup (*n* = 6,170)
Sleep duration (h/night)			
<7	1.01 (0.86, 1.18)	1.01 (0.82, 1.25)	0.97 (0.75, 1.25)
7 to <8	1.00 (ref.)	1.00 (ref.)	1.00 (ref.)
8 to <9	1.04 (0.95, 1.13)	1.01 (0.90, 1.13)	1.07 (0.93, 1.23)
≥9	**1.25 (1.14, 1.38)**	**1.22 (1.08, 1.38)**	**1.30 (1.11, 1.52)**
Bedtime (p.m.)			
≤9:00	**1.27 (1.11, 1.45)**	**1.32 (1.11, 1.56)**	1.21 (0.98, 1.51)
>9:00 to 10:00	**1.16 (1.08, 1.26)**	**1.15 (1.04, 1.27)**	**1.18 (1.04, 1.34)**
>10:00 to 11:00	1.00 (ref.)	1.00 (ref.)	1.00 (ref.)
>11:00	0.98 (0.87, 1.09)	1.00 (0.86, 1.16)	0.94 (0.78, 1.12)

Model adjusted for age, gender, body mass index, education level, smoking status, drinking status, regular exercise, sleep quality, hypertension, diabetes, hyperlipidemia, taking ototoxic drug, and occupational noise exposure (not in ARHL/NIHL subgroup analyses).

We further present the joint associations of sleep duration and bedtime with hearing loss in Fig. S3. Compared with a sleep duration of 7 to <8 h/night and bedtime >10:00 p.m. to 11:00 p.m., participants whose sleep duration ≥9 h/night and bedtime ≤10:00 p.m. had the highest risk of hearing loss with a significant estimated OR (95% CIs) of 1.32 (1.17, 1.48) in the overall population. Similar results were also observed in the ARHL and NIHL subgroups.

In addition, we assessed the associations of sleep quality and midday napping with hearing loss, but no significant relationships were observed (Table S3). We conducted sensitivity analyses for associations of sleep duration and bedtime with hearing loss after excluding those who had cancer, cardiovascular disease, and stroke. As displayed in Table S4, sleeping ≥9 h/night was still related to hearing loss in the overall population, the ARHL subgroup, and the NIHL subgroup, compared to sleeping 7 to <8 h/night. The positive relationships of hearing loss with bedtime ≤9:00 p.m. and >9:00 p.m. to 10:00 p.m. (vs. >10:00 p.m. to 11:00 p.m.) were robust for the overall population, the ARHL subgroup, and the NIHL subgroup (Table S4). Considering the impact of using other medications, such as hypnotics, we conducted primary models with adjustment added use of hypnotics, and the significant relationships of longer sleep duration and early bedtime with hearing loss were still observed in the overall population, the ARHL subgroup, and the NIHL subgroup (Table S5). After adjusting for stroke, we found the positive relationships of sleeping ≥9 h/night and bedtime ≤9:00 p.m. and >9:00 p.m. to 10:00 p.m. with hearing loss in the overall population, the ARHL subgroup, and the NIHL subgroup (Table S6).

### Association of PRS with hearing loss

Table S1 shows detailed information on the 37 hearing loss-related SNPs in the DFTJ cohort. The sets of 37 hearing loss-related SNPs explained 15.21% of phenotypic variance. The weighted PRS was 34.4 ± 4.4 in the overall population (Table 1). Table 3 displays the relationships of hearing loss with PRS as a continuous or categorical variable according to its tertile. Among the overall population, each 5-risk allele increase in PRS was associated with an elevated risk of 29% (95% CI, 24%, 34%) for hearing loss. Compared with the lowest tertile of PRS, the medium and highest tertile of PRS were related to increased risk of hearing loss with an estimation of 23% (95% CI, 13%, 34%) and 67% (95% CI, 53%, 82%), respectively. Meanwhile, we also found positive relationships between hearing loss and PRS among the ARHL subgroup and the NIHL subgroup. Similar results remained significant for PRS calculated by 8 SNPs (Table S7).

**Table 3.** Associations of PRS with hearing loss.

Population	PRS				
	Continuous ^a^	Low	Medium	High	*P* trend
Overall (*n* = 15,827)	**1.29 (1.24, 1.34)**	1.00 (ref.)	**1.23 (1.13, 1.34)**	**1.67 (1.53, 1.82)**	<0.001
ARHL subgroup (*n* = 9,456)	**1.28 (1.21, 1.34)**	1.00 (ref.)	**1.25 (1.12, 1.39)**	**1.65 (1.48, 1.85)**	<0.001
NIHL subgroup (*n* = 6,170)	**1.30 (1.22, 1.38)**	1.00 (ref.)	**1.21 (1.06, 1.39)**	**1.69 (1.48, 1.94)**	<0.001

^a^ The OR (95% CI) of hearing loss when PRS increased 5 risk alleles. Model adjusted for age, gender, body mass index, education level, smoking status, drinking status, regular exercise, sleep quality, hypertension, diabetes, hyperlipidemia, taking ototoxic drug, and occupational noise exposure (not in ARHL/NIHL subgroup analyses).

### Joint associations of sleep duration and PRS or bedtime and PRS with hearing loss

**Fig. 2:**
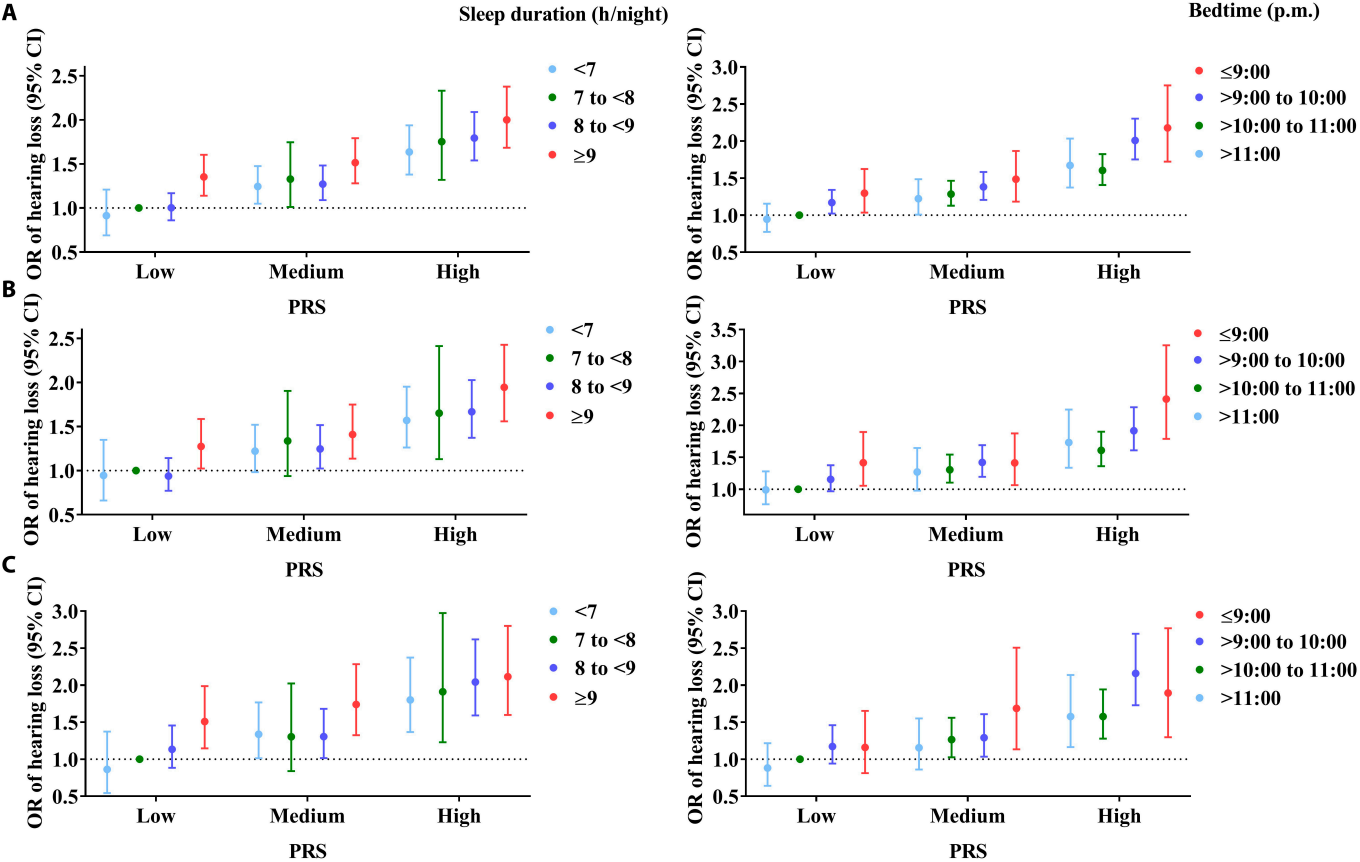
Joint associations of sleep duration and PRS, or bedtime and polygenic risk score with hearing loss in the overall population (A), the ARHL subgroup (B), and the NIHL subgroup (C). References were set at the low level of PRS and sleep duration of 7 to <8 h/night, or the low level of PRS and bedtime of >10:00 p.m. to 11:00 p.m. Model adjusted for age, gender, body mass index, education level, smoking status, drinking status, regular exercise, sleep quality, hypertension, diabetes, hyperlipidemia, taking ototoxic drug, and occupational noise exposure (not in ARHL/NIHL subgroup analyses).

As presented in Fig. 2A, compared with a sleep duration of 7 to <8 h/night and low PRS, the risk of hearing loss showed increased magnitude with an incremental category of sleep duration and PRS. For instance, the ORs (95% CI) of hearing loss were 1.27 (1.09, 1.48) for sleeping 8 to <9 h/night and medium PRS, 1.51 (1.28, 1.79) for sleeping ≥9 h/night and medium PRS, and 2.00 (1.68, 2.38) for sleeping ≥9 h/night and high PRS when setting sleeping 7 to <8 h/night and low PRS as reference. A similar joint association was observed when bedtime and PRS were considered together in the overall population (Fig. 2A). Compared to bedtime >10:00 p.m. to 11:00 p.m. and low PRS, the effect on the risk of hearing loss was elevated with increased bedtime and PRS. Such relationship was the strongest in those who had bedtime ≤9:00 p.m. and high level of PRS with an estimated OR (95% CI) of 2.18 (1.72, 2.75).

Similar joint relationships of sleep duration and PRS, or bedtime and PRS with hearing loss were found in the ARHL subgroup and the NIHL subgroup (Fig. 2B and Fig. 2C). The estimation effect size on hearing loss also displayed an increasing trend with the incremental category of PRS. For example, compared to those with a sleep duration of 7 to <8 h/night and low PRS, the ORs (95% CI) of hearing loss were 1.95 (1.56, 2.43) and 2.11 (1.60, 2.80) for sleeping ≥9 h/night and high PRS in the ARHL subgroup and NIHL subgroup, respectively. Additionally, based on 8 SNPs, the joint association of sleep duration and PRS, or bedtime and PRS with hearing loss were also significant in the overall population, the ARHL subgroup, and the NIHL subgroup (Fig. S4).

### Interactions between sleep duration and PRS or bedtime and PRS on hearing loss

As mentioned above, the independent and joint relationships of sleeping ≥9 h/night and bedtime ≤10:00 p.m. with hearing loss presented significance (Table 2 and Fig. S3). Therefore, we further displayed interactions between sleep duration and PRS on the risk of hearing loss among those with bedtime ≤10:00 p.m. in Table 4, and interactions between bedtime and PRS on the risk of hearing loss among those with sleeping ≥9 h/night in Table 5. Among those with bedtime ≤10:00 p.m., we observed a significant interaction between PRS and sleep duration on hearing loss in the overall population (*P*_int_ = 0.013). For high PRS, sleeping 8 to <9 h/night and ≥9 h/night were associated with hearing loss, compared with sleeping 7 to <8 h/night (Table 4). Similar results were found in the NIHL group, but the interaction of PRS and sleep duration did not show significance (Table 4). Meanwhile, among those with sleeping ≥9 h/night, the interaction between PRS and bedtime on hearing loss appeared significant in the NIHL subgroup (*P*_int_ = 0.017), and risk of hearing loss was related to bedtime ≤10:00 p.m. when compared with >10:00 p.m. to 11:00 p.m. (Table 5). Similar relationships were also observed in the overall population with sleeping ≥9 h/night, but there was no significant interaction between PRS and bedtime (Table 5). Additionally, the above interaction of PRS calculated by 8 SNPs with sleep duration in the overall population and with bedtime in the NIHL subgroup was still robust (*P*_int_ = 0.018 and *P*_int_ = 0.001, respectively; Tables S8 and S9).

**Table 4.** Interactions between sleep duration and PRS on the risk of hearing loss among those with bedtime ≤10:00 p.m.

Sleep duration (h/night)		PRS						*P*_int_
		Low		Medium		High		
		Case/total	OR (95% CI)	Case/total	OR (95% CI)	Case/total	OR (95% CI)	
Overall (*n* = 6,934)								**0.013**
	7 to <8	96/179	1.00 (ref.)	124/208	1.00 (ref.)	119/215	1.00 (ref.)	
	8 to <9	499/1,080	0.78 (0.55, 1.10)	488/969	0.77 (0.55, 1.08)	623/1,026	**1.54 (1.11, 2.12)**	
	≥9	573/1,055	1.07 (0.76, 1.51)	634/1,130	0.97 (0.70, 1.36)	684/1,072	**1.60 (1.16, 2.22)**	
ARHL subgroup (*n* = 4,170)								0.093
	7 to <8	57/106	1.00 (ref.)	72/122	1.00 (ref.)	71/123	1.00 (ref.)	
	8 to <9	290/661	0.65 (0.42, 1.02)	297/609	0.74 (0.48, 1.15)	356/614	1.37 (0.90, 2.09)	
	≥9	320/607	0.93 (0.60, 1.47)	377/690	0.92 (0.59, 1.43)	396/639	1.45 (0.95, 2.21)	
NIHL subgroup (*n* = 2,660)								0.226
	7 to <8	36/70	1.00 (ref.)	48/81	1.00 (ref.)	46/88	1.00 (ref.)	
	8 to <9	201/410	1.19 (0.68, 2.07)	177/345	0.83 (0.49, 1.41)	255/397	**1.86 (1.10, 3.14)**	
	≥9	240/433	1.54 (0.87, 2.70)	244/423	1.14 (0.68, 1.93)	270/413	**1.86 (1.10, 3.16)**	

Model adjusted for age, gender, body mass index, education level, smoking status, drinking status, regular exercise, sleep quality, hypertension, diabetes, hyperlipidemia, taking ototoxic drug, and occupational noise exposure (not in ARHL/NIHL subgroup analyses).

**Table 5.** Interactions between bedtime and PRS on the risk of hearing loss among those with sleep duration ≥9 h/night.

Bedtime (p.m.)		PRS						*P*_int_
		Low		Medium		High		
		Case/total	OR (95% CI)	Case/total	OR (95% CI)	Case/total	OR (95% CI)	
Overall (*n* = 4,038)								0.263
	≤10:00	577/1,062	1.16 (0.85, 1.58)	609/1,082	1.01 (0.73, 1.40)	705/1,113	**1.51 (1.10, 2.08)**	
	>10:00 to 11:00	111/273	1.00 (ref.)	109/260	1.00 (ref.)	108/248	1.00 (ref.)	
ARHL subgroup (*n* = 2,382)								0.910
	≤10:00	321/609	1.27 (0.85, 1.90)	356/661	1.16 (0.75, 1.79)	407/666	1.24 (0.82, 1.88)	
	>10:00 to 11:00	64/169	1.00 (ref.)	54/140	1.00 (ref.)	65/137	1.00 (ref.)	
NIHL subgroup (*n* = 1,596)								**0.017**
	≤10:00	243/438	1.00 (0.60, 1.65)	232/405	0.88 (0.53, 1.46)	279/426	**2.19 (1.29, 3.70)**	
	>10:00 to 11:00	47/102	1.00 (ref.)	54/118	1.00 (ref.)	39/107	1.00 (ref.)	

Model adjusted for age, gender, body mass index, education level, smoking status, drinking status, regular exercise, sleep quality, hypertension, diabetes, hyperlipidemia, taking ototoxic drug, and occupational noise exposure (not in ARHL/NIHL subgroup analyses).

### Subgroup analyses

**Fig. 3.**
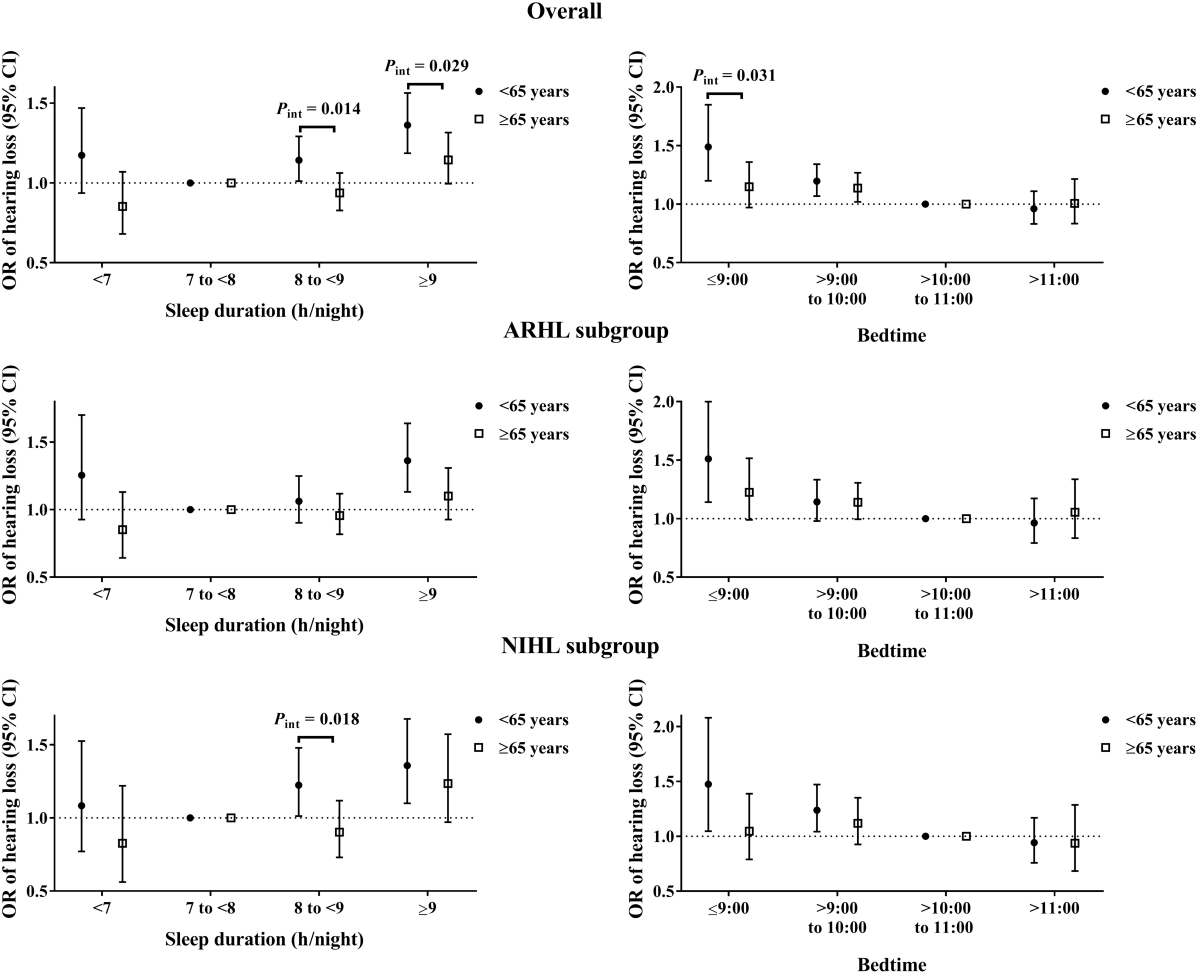
Associations of sleep duration and bedtime with hearing loss stratified by age in the overall population, the ARHL subgroup, and the NIHL subgroup. Model adjusted for age, gender, body mass index, education level, smoking status, drinking status, regular exercise, sleep quality (good, fair, or poor), hypertension, diabetes, hyperlipidemia, taking ototoxic drug, and occupational noise exposure.

Associations of sleep duration and bedtime with hearing loss stratified by major characteristics among the overall population, the ARHL group, and the NIHL group are displayed in Fig. 3 and Figs. S5 to S7. Only the modification of age on relationships of sleep duration and bedtime with hearing loss showed significance (*P*_int_ < 0.05); such relationships were more evident in participants aged <65 years than in those aged ≥65 years among the overall population and the NIHL group (Fig. 3). However, we did not find a significant pair interaction of age and sleep duration, and age and bedtime with hearing loss in the ARHL group (Fig. 3). We found a consistent stronger association of sleep duration and bedtime with hearing loss among those with BMI ≥24 kg/m^2^, diabetes, and hyperlipidemia, but a modification of these characteristics did not show significance in the overall population (Fig. S5). There were more evident associations of sleep duration and bedtime with hearing loss in male, BMI <24 kg/m^2^, and diabetes among the ARHL subgroup, while the above relationships were more obvious in male, BMI ≥24 kg/m^2^, and hyperlipidemia among the NIHL subgroup, all of which did not show significant interactions (Figs. S6 and S7).

## Discussion

In the present study, long sleep duration (≥9 h/night) and early bedtime (≤10:00 p.m.) were associated with nearly 16% to 27% elevated risk of hearing loss compared to recommended sleeping patterns. Meanwhile, PRS was dose–responsively linked to hearing loss. The combination of sleep duration and bedtime with PRS conferred increased risk of hearing loss. Also, a significant interaction of PRS and sleep duration in those within bedtime ≤10:00 p.m. and an interaction of PRS and bedtime in those within sleeping ≥9 h/night were observed, indicating that those with greater genetic risks might be more susceptible to the impact of sleep duration and bedtime on hearing loss. Similarly, the above relationships were also observed in the ARHL and NIHL subgroups. Taken together, our results add to important knowledge of population-based prevention strategies for hearing loss.

Previous studies available on the relationship between sleep and hearing loss were quite limited and inconsistent [8–10]. A study based on hearing loss at a single frequency including 1 kHz or 4 kHz showed that a higher prevalence of hearing loss was associated with sleep duration ≥8 h/night, compared with 6 h/night or ≤5 h/night in Japanese adults [10]. Another cross-sectional study observed a positive association between sleep duration ≥8 h/night and high-frequency hearing loss in American adults, but only significant in those without self-reported occupational noise exposure, while such a relationship was inverse in thousands of Chinese adults, which was significant in those with self-reported occupational noise exposure [9]. However, a US cross-sectional study did not find any significant effect of sleep duration on hearing loss in 632 older people [8]. Based on the large sample size and hearing loss at a wide range of frequencies, the present study showed a consistent association between sleep duration ≥9 h/night and increased risk of hearing loss when compared with the recommended sleep duration of 7 to <8 h/night. Our results threw scientific evidence that longer sleep should be paid more attention to preventing and controlling the development of hearing loss.

Meanwhile, time of sleep onset also yielded a significant influence on circadian rhythm, which could increase the risk of multiple diseases, including cardiovascular and nervous system disorders [7,17]. A previous cohort study conducted in multiple countries showed that bedtimes earlier than 10:00 p.m. or later than midnight might be a risk for all-cause mortality and major cardiovascular events [18]. However, to the best of our knowledge, this is the first study to assess the relation between bedtime and hearing loss. We found an increased risk of hearing loss in overall participants with bedtime ≤10:00 p.m. when compared with bedtime >10:00 p.m. to 11:00 p.m., but not in those having bedtime >11:00 p.m., which might be due to the fact that 40.2% of people with late bedtime had an appropriate sleep duration. Accordingly, prospective studies were needed to further verify our results with more objective documented sleeping information.

Furthermore, hereditary susceptibility was also an important factor in the development of hearing loss. Several studies devoted efforts to exploring the relationships of hearing loss with single SNPs based on GWAS results [12,19,20] or several genetic loci [21,22]. To date, only a cross-sectional study from Europe conducted the calculation of PRS to predict self-reported hearing loss, but it did not consider the number of increased alleles in PRS [23]. To clump the effect of a single SNP, this study calculated the weighted PRS based on 37 hearing loss-related genetic loci from a large-scale cohort study [12]. Although the single effects of some SNPs on hearing loss did not show significance, the association of PRS from these SNPs and hearing loss was robust and in a dose–response manner.

Both genetic risk and lifestyles were regarded as contributing to the etiology of complex diseases, while the effect of lifestyles might be different depending on inherited genetic susceptibility [24,25]. A cross-sectional study observed the significant joint association of sleep and diabetes-related PRS with type 2 diabetes in Americans [26]. However, for all we know, the present study firstly explored whether there were joint effects of PRS with sleep duration and bedtime on hearing loss. Results showed that the combined effects of PRS and sleep duration or PRS and bedtime were stronger than the individual effects of each factor on hearing loss. That is, our study underscored that ignoring genetic predisposition might underestimate hearing loss risk for prevention efforts. Besides, we found the modification of PRS on the associations of sleep duration and bedtime with hearing loss. Notably, the effects of sleep duration and bedtime on hearing loss were only significant in high PRS and not in low and medium PRS. However, we could not directly compare our findings with previous data, as no similar studies were reported. All these findings indicated that if the association hypothesis held, except for switching to recommended sleep patterns, identification of high genetic risk populations would be of great importance for the prevention and control of hearing loss.

It is acknowledged that aging and noise are the most important risk factors for hearing loss, resulting in ARHL and NIHL separately [27], which have a different pathogenesis. ARHL was developed with the natural aging of the auditory system [15], while NIHL was due to the cumulative effect of noise exposure [28]. Based on detailed information on noise exposure in middle-aged and older people, we also found that the positive relationships of longer sleep duration and earlier bedtime with ARHL and NIHL, and its genetic predisposition were significant. With limited evidence supporting our results, a cross-sectional study observed that the association of long sleep duration and high-frequency hearing loss was more evident in American adults with self-reported occupational noise exposure [9]. It could be interpreted that noise exposure might result in a reduction of sleep quality, and even longer sleep duration, which were related to an increased risk of hearing loss.

The underlying mechanisms behind associations of sleep duration and bedtime with hearing loss have not been documented. The development of hearing loss attributed to inner ear homeostasis was relevant to microvascular damage [29], which was reported to be associated with longer sleep duration in our prior studies [18,30]. Long sleep duration or early bedtime also showed a positive relation with nervous system impairment, systemic inflammation, and oxidative stress [31,32]. It is reported that the elevated NLRP3 inflammasome, cytokines, and reactive oxygen species could activate macrophages or autophagy pathways, disrupt the endothelial cell integrity of the stria vascularis and increase hair cell apoptosis, and contribute to hearing loss [27,33,34]. Also, prolonged sleep duration was likely to result from sleep-disordered breathing, which would reduce blood oxygen saturation of inner ear microvessels with elevated inflammation response, and it is reported that moderate and severe sleep apneas were related to high-frequency hearing loss [35]. Additionally, a recent experiment in vivo observed that irregular biological clocks could reduce outer hair cells and synaptic ribbons, enhance oxidative stress, and augment permanent threshold shifts for NIHL mice, indicating that early or late bedtime might have a potential influence on hearing loss [36]. Accordingly, more experiments in vivo were needed to explore the underlying mechanism behind the associations between sleep pattern and hearing loss in the future.

In addition, significant interactions were observed between age and sleep duration or bedtime, and the higher risk of hearing loss was more likely in those aged <65 years than in those aged ≥65 years in the overall population and the NIHL subgroup. In the present study, the percentage of occupational noise exposure in subjects aged <65 years (56.7%) was evidently larger compared to their counterparts (43.3%) in the overall population. Previous studies including ours showed that noise exposure was related to a higher risk of hearing loss [14]. Further excluding those who had occupational noise exposure, the modification of age on association of sleep duration and bedtime with hearing loss in the ARHL subgroup did not show significance. Therefore, although participants aged ≥65 years had a slightly longer sleep duration (8.3 ± 1.0 h/night) than those aged <65 years (8.1 ± 1.0 h/night) in the present study, we believe that a different occupational noise exposure would play a more important role for varied impact on hearing loss between the 2 age groups. In addition, older people generally had multiple risk factors, such as hypertension, diabetes, and hyperlipidemia, which might mask or attenuate the effect of sleep patterns on hearing loss in participants aged ≥65 years, although we have adjusted these risk factors. We also found consistently stronger relationships of sleep duration and bedtime with hearing loss in those who had BMI ≥24 kg/m^2^, diabetes, or hyperlipidemia, although there was no interaction. Previous studies reported that obesity [37], diabetes [38], and hyperlipidemia [39] were associated with an elevated risk of hearing loss, which might support the idea that the effects of sleep patterns on hearing loss would be amplified.

The present study was the largest sample size investigation available on the associations of sleep patterns with hearing loss taking polygenetic risk into account. However, some limitations also needed to be addressed. Firstly, given the limits of causal interpretation with a cross-sectional design, the interpretation of our findings needed to be based on solid assumptions. Secondly, information on sleep duration and bedtime was collected from the questionnaire; thus, sleep duration reflects time spent in bed attempting to sleep, but not the actual sleep duration. It might be more accurate according to the recording of biological sleep, but it was hard to measure the sleep duration and bedtime of each participant with the specific device in large prospective population studies. Although sleep information was only collected for the recent 6 months, participants were retirees from Dongfeng Motor Corporation, who have a relatively fixed sleep schedule. Thirdly, history of having sudden deafness and traumatic deafness was also acquired from the semi-structured questionnaire, which might have misclassification bias. We also could not identify auditory neuropathy from hearing loss based on PTA, because tests for brainstem auditory evoked potentials and the function of outer hair cells for auditory neuropathy are difficult to apply in large-scale cohort studies. Fourthly, different races have different genetic susceptibility, whereas the SNPs used to calculate PRS in the present study were selected from the GWAS meta-analysis study in the Western population but not Chinese. Fifthly, hearing loss due to gene mutation is likely to affect the assessment of associations between sleep patterns and hearing loss, although we still found the significant positive associations of longer sleeping (>9 h/night) and early bedtime (≤9:00 p.m.) with hearing loss in those with low genetic risk. Sixthly, although multiple confounders have been considered in our analyses, other potential covariates might not be available in the present study, such as daytime sleep duration, snoring, sleep apnea, and psychological factors. Although we minimized external distractions when taking audiometric tests, psychological factors could not be avoided to affect results. Lastly, the results came from middle-aged and old people; thus, one should be cautious before generalizing to populations of all ages and other regions.

In conclusion, longer sleep duration and earlier bedtime were independently and in combination associated with an increased risk of hearing loss, which was enhanced at high genetic predisposition. Such relationships were also significantly observed for ARHL and NIHL. Our results underscored the importance of paying attention to both genetics and sleep pattern for risk assessment of hearing loss, particularly for those with high genetic risk. More rigorous prospective studies or randomized controlled trials are warranted to verify our findings in the future.

## Methods

### Study design and subjects

The DFTJ cohort study recruited retirees of Dongfeng Motor Corporation in Shiyan City, Hubei province, China, which were described in detail previously [16]. A total of 38,295 retirees participated in the survey in 2013, and all individuals were required to finish a semi-structured questionnaire, health examinations, and fasting blood drawing.

As shown in Fig. S1, after excluding lack of audiometric tests (*n* = 18,479), sudden deafness (*n* = 725), traumatic deafness (*n* = 36), those with missing sleep information (*n* = 251), and other covariates in the statistic model (*n* = 320), 18,484 individuals had both valid audiometric data and sleep-related information. Further excluding those who lacked genotype (*n* = 1,445), failed GWAS quality controls (*n* = 791), and showed domestic relationships based on pair Identity-By-Descent and PI_HAT according to the previous study [40] (*n* = 421), 15,827 individuals were included in our analyses. Participants who had occupational noise exposure (normalized continuous A-weighted sound pressure level equivalent to 8 h per day of 80 dB [A] or above) (*n* = 6,170) were included for the NIHL analysis. Of the rest of the population, we further excluded those who had missing information on noise exposure (*n* = 21), or conducted hearing loss (*n* = 180), resulting in the remaining 9,456 participants for the ARHL subgroup. The present study was approved by the Medical Ethics Committee of Tongji Medical College and Dongfeng General Hospital. We obtained written informed consent from each participant.

### Audiometric tests

As described detailedly in our previous study [41], trained professional staff employed a Micro-DSP ZD-21 audiometer (Sichuan Micro-DSP Technology Co, Ltd, China) to take audiometric tests for each participant in a sound-isolating room of the Dongfeng Motor Corporation-owned hospital. Pure tone audiometry was measured for the audiometric threshold of each ear at 0.5, 1, 2, 4, and 8 kHz, and each frequency contained an intensity range of −10 to 120 dB. If the participant did not respond at the maximum value, he or she would be regarded as the maximum value. If audiometric threshold was below zero, it was regarded as invalid measurements. Hearing loss was identified as an audiometric threshold with an average value of 25 dB or higher level at 0.5, 1, 2, and 4 kHz in the better ear according to the definition of the World Health Organization [42]. We displayed the distribution of the audiometric threshold of each ear at 0.5, 1, 2, and 4 kHz in Fig. S2.

### Evaluation of sleep duration and bedtime

Information on sleep duration and bedtime has been described elsewhere [43]. Briefly, sleep duration was calculated based on the following question: “What time did you usually go to sleep at night and wake up in the morning over the past 6 months?” Sleep duration was classified as <7 h/night, 7 to <8 h/night, 8 to <9 h/night, and ≥9 h/night, and bedtime was categorized as ≤9:00 p.m., >9:00 p.m. to 10:00 p.m., >10:00 p.m. to 11:00 p.m., and >11:00 p.m. Sleep quality was obtained by the following questionnaire responses: good, fair, and poor. Midday napping was evaluated by asking “Did you have a habit of midday napping over the past 6 months?” We divided midday napping into 4 groups, including no napping (0 min), >0 to 30 min, >30 to 60 min, and >60 min.

### Genotyping and calculation of PRS for hearing loss

Information on GWAS genotypes was described in detail elsewhere [44]. Briefly, all participants were genotyped using Illumina Infinium OminZhongHua-8 chips and imputed the missing genotypes with the reference panel of 3,931 East Asian samples from the 1000 Genomes Project and the SG10K Project within the DFTJ cohort. Hearing loss-related SNPs were identified from the largest and most recently published genome-wide association meta-analysis study [12], of which 37 SNPs reached the quality (minor allele frequency > 0.05 and Hardy–Weinberg equilibrium *P* < 10^−6^) in the present study, and are listed as Table S1.

According to the previously reported weighted method, the weighted PRS was calculated based on the following equation: weighted PRS = [Ln (OR1) × SNP1 + Ln (OR2) × SNP2 + … + Ln (OR37) ×SNP37] × [37/sum of Ln (OR)] [45]. Based on the number of risk alleles, each SNP was recorded as 0, 1, or 2. The coefficient of each SNP on hearing loss from the above GWAS meta-analysis study only focuses on Europeans and Americans [12], while these tag SNPs identified in individuals of European and American descent may not represent Chinese ancestry. Thus, the adopted OR was the coefficient of each SNP on hearing loss from the current study, which was based on GWAS chips and employed logistic models with adjustment for age, gender, and the first 10 principal components. The first 10 principal components were obtained in the overall population after conducting linkage disequilibrium pruning. The weighted PRS ranges from 0 to <74, with higher scores indicating a higher genetic risk of hearing loss.

### Assessment of covariates

Semi-structured questionnaires were designed to obtain the basic information of retirees by trained interviewers, including sociodemographic characteristics (age, gender, and education level), lifestyle (sleep, smoking status, drinking status, and regular exercise), and personal and family medical histories. According to the workplace and the monitoring records for each participant, occupational noise exposure refers to those who were exposed to normalized continuous A-weighted sound pressure level equivalent to 8 h per day of 80 dB (A) or above (L_Aeq, 8h_ ≥ 80 dB [A]) for no less than 1 year [46]. More descriptions are provided in the Supplementary Materials.

### Statistical analysis

Participants’ characteristics were summarized as means ± standard deviation, median (interquartile range), or numbers (percentages) according to their distributions. We compared the differences between the ARHL subgroup and the NIHL subgroup by Student’s *t*-test for continuous variables and the chi-square test for the categorical variables. We employed multivariable logistic regression models to assess the associations of sleep duration and bedtime with hearing loss in the overall population, the ARHL subgroup, and the NIHL subgroup. Sleeping 7 to <8 h/night and bedtime >10:00 p.m. to 11:00 p.m. were set as reference groups based on the suggestion in prior studies [6,7]. Based on previous studies and statistical considerations (lowest Akaike Information Criterion) [8,41], models were adjusted for age, gender, BMI, and education levels (primary school or below, middle school, high school, or beyond), smoking status (current, ever, or never), drinking status (current, ever, or never), regular exercise (yes or no), sleep quality (good, fair, or poor), hypertension (yes or no), hyperlipidemia (yes or no), diabetes (yes or no), taking ototoxic drug (yes or no), and occupational noise exposure (yes or no) only in the overall population analysis but not in ARHL/NIHL subgroup analyses. We conducted a restricted cubic spline to examine the dose–response relationship of sleep duration and hearing loss. Knots were placed at the 25th, 50th, and 75th percentiles of sleep duration distribution, and the reference was set at 7.5 h/night. We also analyzed the joint association of sleep duration, bedtime, and hearing loss. Meanwhile, we performed multivariable logistic regression to evaluate the relationships of PRS and hearing loss as the above primary models.

Further, we explored the joint associations or interactions of sleep duration and PRS, and bedtime and PRS with hearing loss. The joint association of sleep duration and PRS, and bedtime and PRS on hearing loss was conducted based on the following reference: the low level of PRS and sleeping 7 to <8 h/night, and the low level of PRS and bedtime >10:00 p.m. to 11:00 p.m., respectively. Given the significant joint effect of sleep duration and bedtime on hearing loss (results shown in Fig. S3), the positive association with hearing loss was only significant in those with both sleeping ≥9 h/night and bedtime ≤10:00 p.m. in the overall population, the ARHL subgroup, and the NIHL subgroup. Therefore, we further assessed the interaction of PRS and sleep duration with hearing loss in those whose bedtime ≤10:00 p.m., and the interaction of PRS and bedtime with hearing loss in those sleeping ≥9 h/night. Interaction analyses were conducted by adding multiplied interaction terms sleep duration × PRS and bedtime × PRS in the above multivariable logistic regression models.

In addition, we carried out stratification analyses by the major characteristics, including age, gender, BMI, hypertension, diabetes, and hyperlipidemia, and derived their modification effects on the relationships of sleep duration and bedtime with hearing loss by adding the multiplied interaction terms to the above primary models. We also assessed the associations of sleep quality and midday napping with hearing loss. Finally, we conducted sensitivity analyses for associations of sleep duration and bedtime with hearing loss after excluding those who had cancer, cardiovascular disease, and stroke. Given the impact of sleep medicine, we further carried out the primary model after excluding those who took hypnotics in the recent 2 weeks. Due to the possible influence of stroke on hearing loss, we conducted the primary model with adjustment added stroke. Considering that some SNPs in PRS showed no significance in our GWAS, we further included only 8 SNPs with a significant effect on hearing loss according to Table S1 (*P* < 0.01) and calculated weighted PRSs. We assessed the association of hearing loss with PRSs calculated by 8 SNPs as well as its joint and interactions with sleep patterns.

The SAS program (version 9.4, SAS Institute, Cary, NC), R software (version 4.0.4; R Core Team), or PLINK was applied for analyses, and *P* < 0.05 (2-sided) was regarded as statistically significant.

## Data Availability

All data in the paper are available upon reasonable request.
