# Supplementary material for: Independent and Combined Associations of Sleep Duration, Bedtime, and Polygenic Risk Score with the Risk of Hearing Loss among Middle-Aged and Old Chinese: The Dongfeng–Tongji Cohort Study
Source: Research (Wash D C). 2023 Jun 27;6:0178. doi: 10.34133/research.0178 (PMC10298215; doi:10.34133/research.0178)
Supplement: Supplementary file 1 — Figs. S1 to S7 Tables S1 to S9 [file research.0178.f1.docx]

**Supplementary Material**

**Independent and combined associations of sleep duration, bedtime, and polygenic risk score with the risk of hearing loss among middle-aged and old Chinese: The Dongfeng-Tongji cohort study**

Miao Liu^1^, Haiqing Zhang^1^, Zhichao Wang**^2^**, Tingting Mo^1^, Xuefeng Lai^1^, Yaling He^1^, Minghui Jiang^3^, Meian He^1^, Weijia Kong**^2^**^*^, Tangchun Wu^1*^, Xiaomin Zhang^1*^

^1^ Department of Occupational and Environmental Health, Ministry of Education Key Laboratory of Environment and Health, and State Key Laboratory of Environmental Health (Incubating), School of Public Health, Tongji Medical College, Huazhong University of Science and Technology, Wuhan, Hubei, China.

^2^ Department of Otorhinolaryngology, Union Hospital, Tongji Medical College, Huazhong University of Science and Technology, Wuhan, Hubei, China.

^3^ Department of Epidemiology, School of Public Health, Tongji Medical College, Huazhong University of Science and Technology, Wuhan, Hubei, China.

**Assessment of covariates**

Education level was divided into 3 groups, including primary school or below, middle school, and high school or higher. Those who smoked at least 1 cigarette a day for over half a year were regarded as current smokers. Those who drank at least once a week for over half a year were defined as current drinkers. Former smokers and former drinkers were participants who quit smoking or drinking for more than 6 months, respectively. If participants took regular exercise for more than 30 min and 5 times a week during past 6 months, he or she would be regarded as regular exercise. We calculated body mass index (BMI) as the ratio of weight (kg) to the square of height (m^2^). Participants who had systolic blood pressure ≥140 mmHg or diastolic blood pressure ≥90 mmHg, or self-reported history of hypertension, or taken anti-hypertensive medications were regarded as having hypertension. Participants who had a level of fasting glucose ≥7.0 mmol/L, or self-reported history of diabetes or taken anti-diabetic medications were identified as having diabetes. If total cholesterol ≥5.72 mmol/L, or triglycerides ≥1.70 mmol/L, or with self-reported diagnosed hyperlipidemia or use of lipid-lowering drugs, they were defined as hyperlipidemia. Take ototoxic drug referred to take gentamicin, streptomycin or kanamycin in the past.

**Table of contents**

**Fig. S1.** Flowchart of inclusion and exclusion criteria.

**Fig. S2.** The distribution of audiometric threshold of each ear at 0.5 kHz, 1 kHz, 2 kHz and 4 kHz.

**Table S1.** Associations of previously GWAS reported SNPs with hearing loss.

**Table S2.** Characteristics of sleep duration, bedtime and PRS in subjects with hearing loss and normal hearing.

**Fig. S3.** Joint associations of sleep duration and bedtime with hearing loss in overall (A), ARHL subgroup (B) and NIHL subgroup (C).

**Table S3.** Associations of sleep quality and nap time with hearing loss.

**Table S4.** Associations of sleep duration and bedtime with hearing loss after excluding those who had cancer, cardiovascular disease and stroke.

**Table S5.** Associations of sleep duration and bedtime with hearing loss after excluding those who took hypnotics.

**Table S6.** Associations of sleep duration and bedtime with hearing loss in primary models with adjustment adding stroke.

**Table S7.** Sensitive analyses on associations of PRS calculated by 8 SNPs with hearing loss

**Fig. S4.** Joint associations of sleep duration and PRS, or bedtime and PRS calculated by 8 SNPs with hearing loss in overall (A), ARHL subgroup (B) and NIHL subgroup (C).

**Table S8.** Interactions between sleep duration and PRS calculated by 8 SNPs on the risk of hearing loss among those with bedtime ≤10:00 p.m.

**Table S9.** Interactions between bedtime and PRS calculated by 8 SNPs on the risk of hearing loss among those with sleep duration ≥9 hours/night

**Fig. S5.** Associations of sleep duration and bedtime with hearing loss stratified by major characteristics in overall.

**Fig. S6.** Associations of sleep duration and bedtime with hearing loss stratified by major characteristics in ARHL subgroup.

**Fig. S7.** Associations of sleep duration and bedtime with hearing loss stratified by major characteristics in NIHL subgroup.


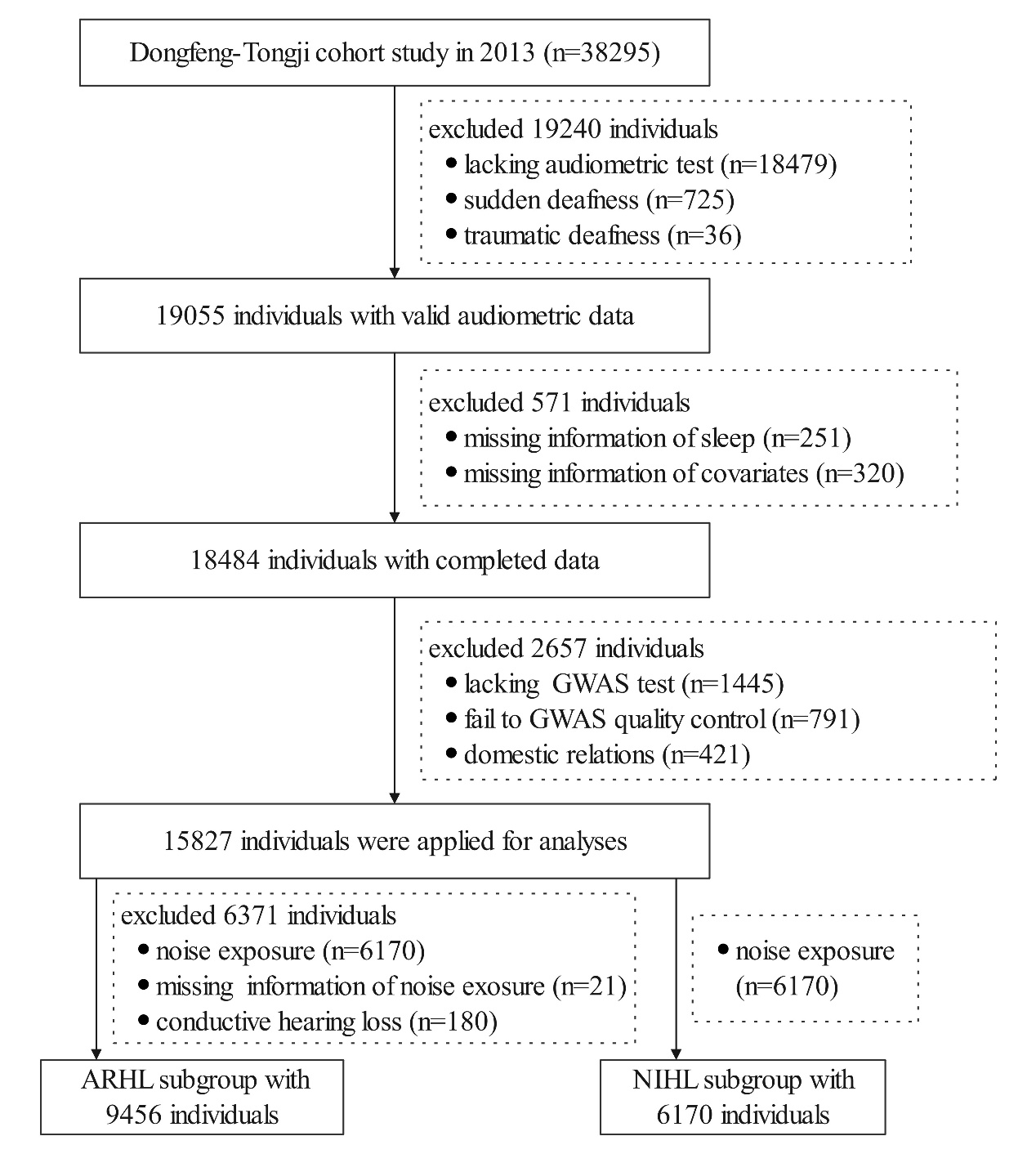


**Fig. S1.** Flowchart of inclusion and exclusion criteria.


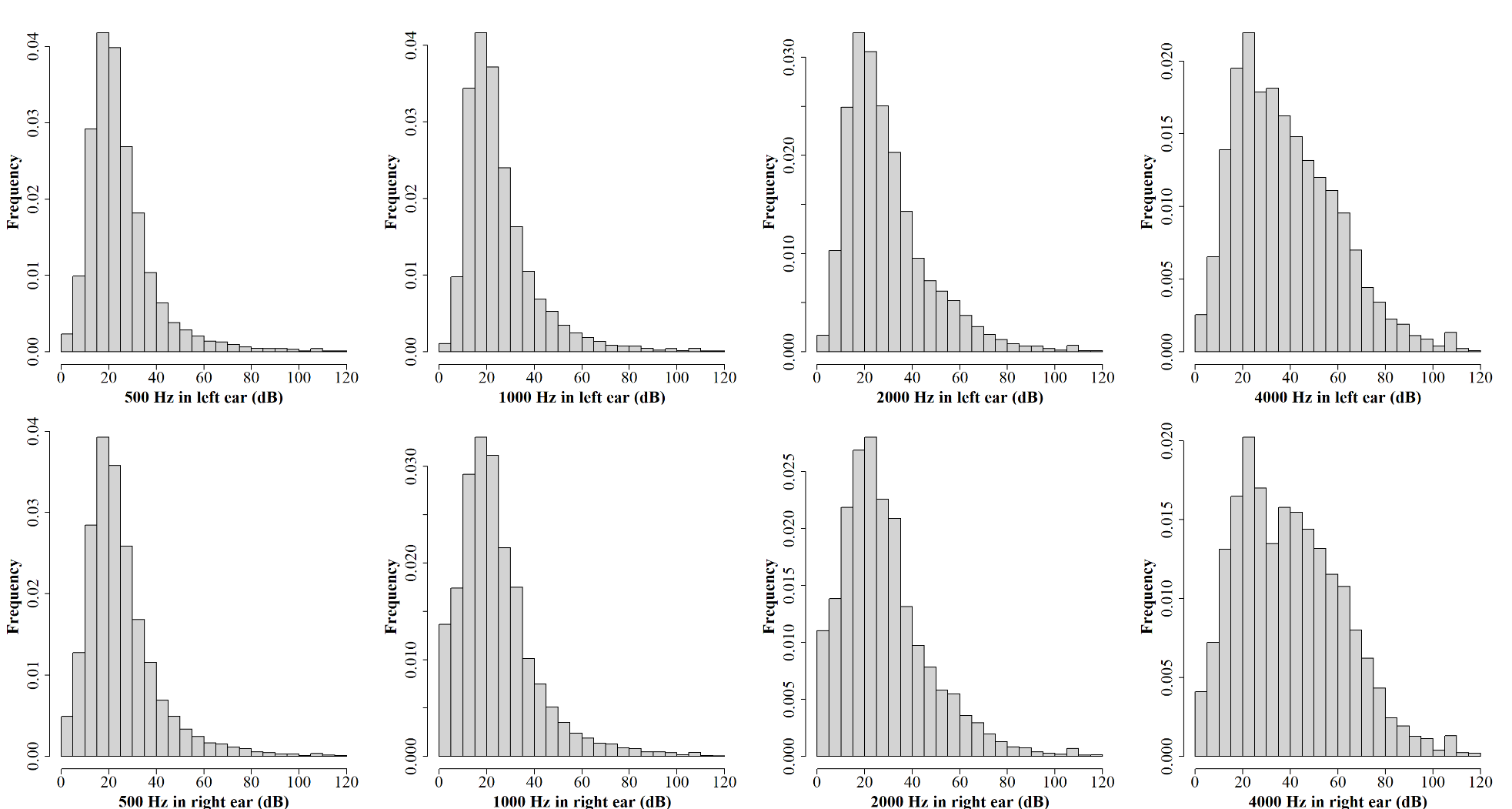


**Fig. S2.** The distribution of audiometric threshold of each ear at 0.5 kHz, 1 kHz, 2 kHz and 4 kHz.

**Table S1.** Associations of previously GWAS reported SNPs with hearing loss

| SNP ^a^ | Chr | BP (GRCh38) | Locus gene | EA | EAF ^b^ | HWE ^b^ | OR ^b^ | *P* ^b^ |
| --- | --- | --- | --- | --- | --- | --- | --- | --- |
| rs4660885 | 1 | 45778084 | *IPP-[x]-MAST2* | G | 0.444 | 0.303 | 1.055 | 4.03E-02 |
| rs7525101 | 1 | 165139894 | *PBX1—[x]-LMX1A* | T | 0.873 | 0.172 | 1.009 | 7.27E-01 |
| rs6545432 | 2 | 54590546 | *SPTBN1* | A | 0.641 | 0.437 | 1.039 | 1.22E-01 |
| rs741475 | 2 | 207222415 | *KLF7-[x]–CREB1* | C | 0.395 | 0.868 | 1.041 | 1.55E-01 |
| rs3915060 | 3 | 121994133 | *ILDR1* | T | 0.707 | 0.954 | 1.002 | 9.56E-01 |
| rs72622585 | 3 | 182274527 | *SOX2—[x]—ATP11B* | T | 0.515 | 0.436 | 1.069 | 9.82E-03 |
| rs13148153 | 4 | 17515935 | *CLRN2* | C | 0.925 | 0.390 | 1.050 | 3.03E-01 |
| rs323693 | 5 | 2562479 | *IRX4—[x]–IRX2* | C | 0.423 | 0.313 | 1.036 | 2.59E-01 |
| rs1981809 | 5 | 73624204 | *UTP15-[x]-ARHGEF28* | C | 0.336 | 0.656 | 1.060 | 2.42E-02 |
| rs4413512 | 5 | 73781524 | *ARHGEF28* | G | 0.486 | 0.824 | 1.110 | 8.60E-05 |
| rs13171669 | 5 | 149221680 | *ABLIM3* | G | 0.627 | 0.838 | 1.047 | 6.22E-02 |
| rs7764856 | 6 | 32712863 | *HLA-DQB1-[x]-HLA-DQA2* | A | 0.367 | 0.472 | 1.074 | 1.13E-02 |
| rs4714678 | 6 | 43374853 | *ZNF318-[x]-ABCC10* | G | 0.607 | 0.934 | 1.047 | 9.94E-02 |
| rs9493627 | 6 | 133468590 | *EYA4* | A | 0.340 | 0.873 | 1.052 | 4.39E-02 |
| rs2296508 | 6 | 158076685 | *SYNJ2* | C | 0.563 | 0.043 | 1.046 | 6.68E-02 |
| rs11238325 | 7 | 50785454 | *GRB10* | T | 0.733 | 0.216 | 1.063 | 2.05E-02 |
| rs4732339 | 7 | 138807094 | *TMEM213-[x]-KIAA1549* | A | 0.794 | 0.680 | 1.039 | 1.34E-01 |
| rs13268718 | 8 | 140677101 | *PTK2* | G | 0.384 | 0.306 | 1.006 | 8.03E-01 |
| rs2393729 | 10 | 62077257 | *ARID5B* | C | 0.557 | 0.253 | 1.049 | 5.88E-02 |
| rs10901863 | 10 | 125123701 | *CTBP2* | T | 0.278 | 0.953 | 1.053 | 1.11E-01 |
| rs7939493 | 11 | 8052063 | *TUB* | A | 0.354 | 0.689 | 1.086 | 4.15E-01 |
| rs566673 | 11 | 66633902 | *RBM14-[x]-RBM4* | T | 0.985 | 1.000 | 1.022 | 4.34E-01 |
| rs67307131 | 11 | 118609508 | *PHLDB1* | C | 0.128 | 0.454 | 1.051 | 9.95E-02 |
| rs7313797 | 12 | 109458360 | *KCTD10* | C | 0.328 | 0.125 | 1.031 | 2.37E-01 |
| rs920701 | 13 | 75842965 | *LMO7* | C | 0.683 | 0.279 | 1.059 | 2.82E-02 |
| rs9517282 | 13 | 98406929 | *FARP1* | C | 0.946 | 0.243 | 1.029 | 3.04E-01 |
| rs1566128 | 14 | 52048263 | *NID2* | A | 0.658 | 0.112 | 1.073 | 6.99E-03 |
| rs4132250 | 15 | 88685769 | *ISG20-[x]–ACAN* | C | 0.316 | 1.000 | 1.088 | 6.26E-04 |
| rs62033400 | 16 | 53777876 | *FTO* | G | 0.358 | 0.604 | 1.014 | 7.21E-01 |
| rs11643684 | 16 | 55456255 | *IRX6–[x]-MMP2* | G | 0.660 | 0.832 | 1.072 | 3.71E-02 |
| rs13337678 | 16 | 56346025 | *GNAO1* | C | 0.277 | 0.439 | 1.018 | 4.58E-01 |
| rs222835 | 17 | 7230810 | *DVL2* | A | 0.380 | 0.354 | 1.025 | 3.41E-01 |
| rs11152089 | 18 | 54958712 | *CCDC68* | T | 0.340 | 0.395 | 1.079 | 6.65E-03 |
| rs11881070 | 19 | 2389142 | *SPPL2B-[x]-TMRPS9* | C | 0.419 | 0.524 | 1.081 | 1.79E-03 |
| rs12980998 | 19 | 4217513 | *ANKRD24* | T | 0.478 | 0.762 | 1.073 | 6.50E-03 |
| rs5756795 | 22 | 37726115 | *TRIOBP* | C | 0.692 | 0.011 | 1.123 | 9.05E-06 |
| rs132931 | 22 | 38091519 | *BAIAP2L2* | G | 0.088 | 0.882 | 1.021 | 4.11E-01 |

^a^ SNP reported in previous GWAS meta-analysis study (Trpchevska et al., 2022) reached MAF>0.05 and HWE>1E-6.

^b^ results obtained in GWAS analyses of present study.

**Table S2.** Characteristics of sleep duration, bedtime and PRS in subjects with hearing loss and normal hearing.

| Variables | Overall | |  | ARHL subgroup | |  | NIHL subgroup | |  |
| --- | --- | --- | --- | --- | --- | --- | --- | --- | --- |
|  | Hearing loss (n=7559) | Normal hearing (n=8268) | *P* | Hearing loss (n=4355) | Normal hearing (n=5101) | *P* | Hearing loss (n=3056) | Normal hearing (n=3114) | *P* |
| Sleep duration, hours/night, n (%) |  |  | <0.001 |  |  | <0.001 |  |  | <0.001 |
| <7 | 409 (5.4) | 501 (6.1) |  | 231 (5.3) | 296 (5.8) |  | 169 (5.5) | 201 (6.5) |  |
| 7 to <8 | 1734 (22.9) | 2215 (26.8) |  | 1011 (23.2) | 1387 (27.2) |  | 693 (22.7) | 811 (26) |  |
| 8 to <9 | 3139 (41.5) | 3620 (43.8) |  | 1810 (41.6) | 2253 (44.2) |  | 1269 (41.5) | 1348 (43.3) |  |
| ≥9 | 2277 (30.1) | 1932 (23.4) |  | 1303 (29.9) | 1165 (22.8) |  | 925 (30.3) | 754 (24.2) |  |
| Bedtime, p.m., n (%) |  |  | <0.001 |  |  | <0.001 |  |  | <0.001 |
| ≤9:00 | 851 (11.3) | 512 (6.2) |  | 500 (11.5) | 319 (6.3) |  | 336 (11.0) | 189 (6.1) |  |
| >9:00 to 10:00 | 3027 (40.0) | 2608 (31.5) |  | 1753 (40.3) | 1629 (31.9) |  | 1202 (39.3) | 967 (31.1) |  |
| >10:00 to 11:00 | 2934 (38.8) | 3846 (46.5) |  | 1693 (38.9) | 2407 (47.2) |  | 1192 (39.0) | 1415 (45.4) |  |
| >11:00 | 747 (9.9) | 1302 (15.8) |  | 409 (9.4) | 746 (14.6) |  | 326 (10.7) | 543 (17.4) |  |
| PRS | 34.9 ± 4.4 | 34.0 ± 4.4 | <0.001 | 34.9 ± 4.4 | 34.0 ± 4.4 | <0.001 | 34.8 ± 4.4 | 34.0 ± 4.4 | <0.001 |

Note: Data were presented as means ± SD for normally distributed variables, median (IQR) for non-normally distributed variables and numbers (percentages) for categorical variables.

Abbreviations: ARHL, age-related hearing loss; NIHL, noise-induced hearing loss; BMI, body mass index; PRS, polygenic risk score; SD, standard deviation; IQR, interquartile range.


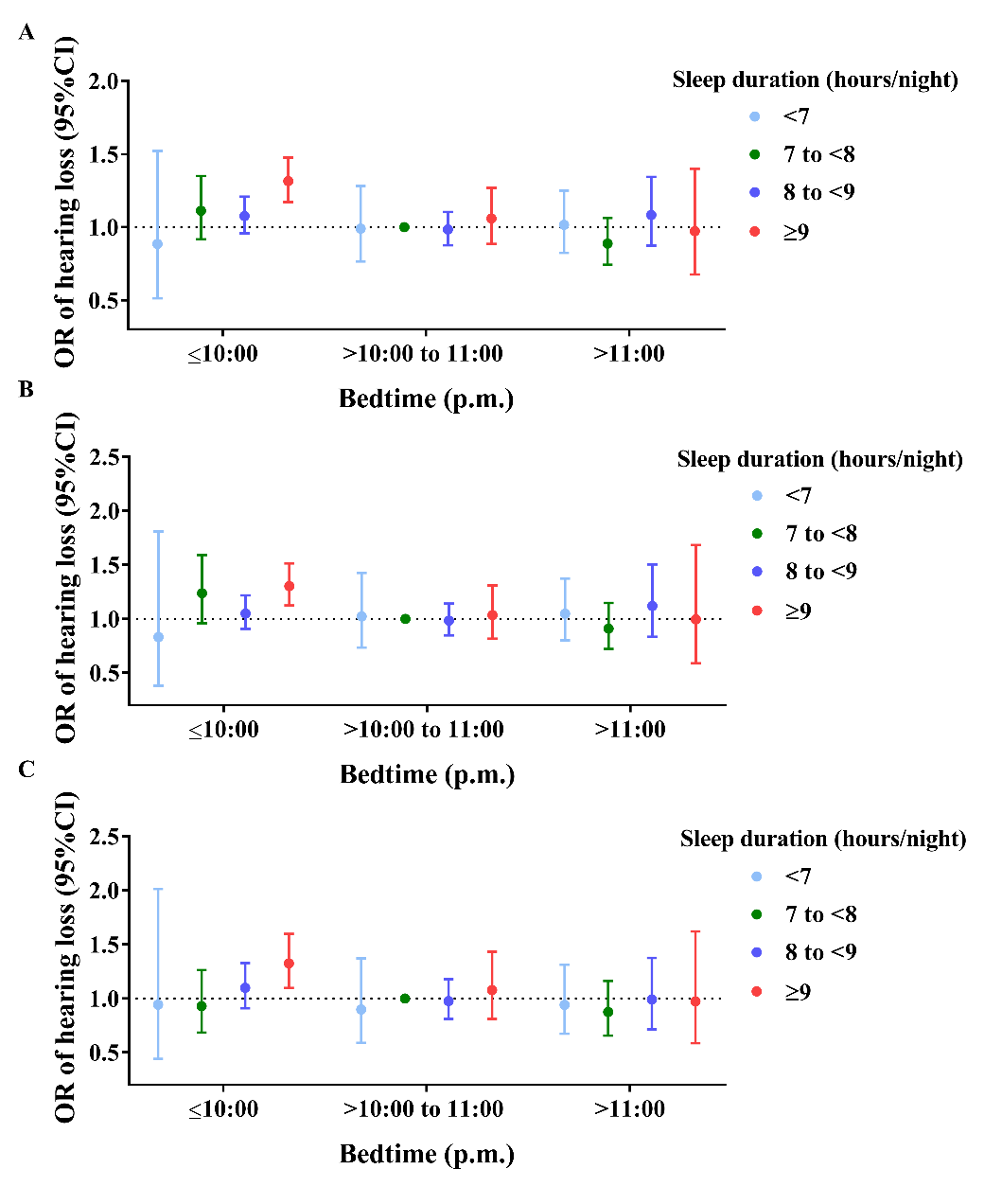


**Fig. S3.** Joint associations of sleep duration and bedtime with hearing loss in overall (A), ARHL subgroup (B) and NIHL subgroup (C). Model adjusted for age, gender, body mass index, education level, smoking status, drinking status, regular exercise, sleep quality, hypertension, diabetes, hyperlipemia, take ototoxic drug and occupational noise exposure (not in ARHL/NIHL subgroup analyses).

**Table S3.** Associations of sleep quality and nap time with hearing loss.

| Variables | Overall  (n=15827) | ARHL subgroup (n=9456) | NIHL subgroup (n=6170) |
| --- | --- | --- | --- |
| Sleep quality |  |  |  |
| Good | 1.00 (ref.) | 1.00 (ref.) | 1.00 (ref.) |
| Fair | 1.02 (0.95, 1.10) | 1.07 (0.97, 1.18) | 0.94 (0.83, 1.07) |
| Poor | 0.97 (0.87, 1.08) | 1.00 (0.87, 1.16) | 0.90 (0.76, 1.07) |
|  |  |  |  |
| Nap time (min) |  |  |  |
| 0 | 1.00 (ref.) | 1.00 (ref.) | 1.00 (ref.) |
| >0 to 30 | 1.03 (0.93, 1.15) | 1.04 (0.91, 1.19) | 1.04 (0.88, 1.23) |
| >30 to 60 | 0.98 (0.88, 1.09) | 0.98 (0.85, 1.13) | 1.01 (0.84, 1.21) |
| >60 | 1.01 (0.89, 1.14) | 0.98 (0.84, 1.16) | 1.10 (0.90, 1.35) |

Model adjusted for age, gender, body mass index and education level, smoking status, drinking status, regular exercise, and sleep quality, hypertension, diabetes, hyperlipemia, take ototoxic drug, occupational noise exposure (not in ARHL/NIHL subgroup analyses).

**Table S4.** Associations of sleep duration and bedtime with hearing loss after excluding those who had cancer, cardiovascular disease and stroke.

| Variables | Overall  (n=12143) | ARHL subgroup (n=7223) | NIHL subgroup (n=4795) |
| --- | --- | --- | --- |
| Sleep duration (hours/night) |  |  |  |
| <7 | 1.01 (0.84, 1.21) | 1.08 (0.85, 1.37) | 0.90 (0.68, 1.20) |
| 7 to <8 | 1.00 (ref.) | 1.00 (ref.) | 1.00 (ref.) |
| 8 to <9 | 1.03 (0.93, 1.13) | 1.00 (0.88, 1.14) | 1.05 (0.90, 1.23) |
| ≥9 | **1.22 (1.09, 1.37)** | **1.20 (1.04, 1.39)** | **1.25 (1.05, 1.49)** |
| Bedtime (p.m.) |  |  |  |
| ≤9:00 | **1.23 (1.05, 1.44)** | **1.32 (1.08, 1.62)** | 1.12 (0.87, 1.44) |
| >9:00 to 10:00 | **1.17 (1.07, 1.28)** | **1.14 (1.01, 1.28)** | **1.20 (1.04, 1.39)** |
| >10:00 to 11:00 | 1.00 (ref.) | 1.00 (ref.) | 1.00 (ref.) |
| >11:00 | 1.02 (0.90, 1.16) | 1.07 (0.91, 1.27) | 0.92 (0.76, 1.12) |

Model adjusted for age, gender, body mass index and education level, smoking status, drinking status, regular exercise, and sleep quality, hypertension, diabetes, hyperlipemia, take ototoxic drug, occupational noise exposure (not in ARHL/NIHL subgroup analyses).**Table S5.** Associations of sleep duration and bedtime with hearing loss after excluding those who took hypnotics.

| Variables | Overall  (n=9841) | ARHL subgroup (n=5892) | NIHL subgroup (n=3718) |
| --- | --- | --- | --- |
| Sleep duration (hours/night) |  |  |  |
| <7 | 1.06 (0.86, 1.31) | 0.99 (0.76, 1.30) | 1.13 (0.81, 1.59) |
| 7 to <8 | 1.00 (ref.) | 1.00 (ref.) | 1.00 (ref.) |
| 8 to <9 | 1.05 (0.94, 1.17) | 0.99 (0.86, 1.15) | 1.12 (0.93, 1.35) |
| ≥9 | 1.34 (1.18, 1.52) | 1.23 (1.04, 1.44) | 1.51 (1.23, 1.86) |
| Bedtime (p.m.) |  |  |  |
| ≤9:00 | 1.27 (1.07, 1.51) | 1.21 (0.98, 1.51) | 1.43 (1.07, 1.92) |
| >9:00 to 10:00 | 1.17 (1.06, 1.30) | 1.16 (1.02, 1.32) | 1.20 (1.01, 1.41) |
| >10:00 to 11:00 | 1.00 (ref.) | 1.00 (ref.) | 1.00 (ref.) |
| >11:00 | 0.97 (0.84, 1.13) | 0.93 (0.76, 1.13) | 1.05 (0.83, 1.32) |

Model adjusted for age, gender, body mass index and education level, smoking status, drinking status, regular exercise, and sleep quality, hypertension, diabetes, hyperlipemia, take ototoxic drug, occupational noise exposure (not in ARHL/NIHL subgroup analyses).

**Table S6.** Associations of sleep duration and bedtime with hearing loss in primary models with adjustment adding stroke.

| Variables | Overall  (n=9841) | ARHL subgroup (n=5892) | NIHL subgroup (n=3718) |
| --- | --- | --- | --- |
| Sleep duration (hours/night) |  |  |  |
| <7 | 1.01 (0.86, 1.18) | 1.02 (0.83, 1.25) | 0.96 (0.75, 1.24) |
| 7 to <8 | 1.00 (ref.) | 1.00 (ref.) | 1.00 (ref.) |
| 8 to <9 | 1.04 (0.95, 1.14) | 1.01 (0.91, 1.13) | 1.08 (0.94, 1.24) |
| ≥9 | **1.26 (1.14, 1.39)** | **1.23 (1.08, 1.39)** | **1.30 (1.11, 1.52)** |
| Bedtime (p.m.) |  |  |  |
| ≤9:00 | **1.27 (1.11, 1.45)** | **1.33 (1.12, 1.58)** | 1.20 (0.96, 1.49) |
| >9:00 to 10:00 | **1.16 (1.08, 1.26)** | **1.15 (1.04, 1.27)** | **1.17 (1.03, 1.33)** |
| >10:00 to 11:00 | 1.00 (ref.) | 1.00 (ref.) | 1.00 (ref.) |
| >11:00 | 0.97 (0.87, 1.09) | 1.00 (0.86, 1.17) | 0.93 (0.78, 1.11) |

Model adjusted for age, gender, body mass index and education level, smoking status, drinking status, regular exercise, and sleep quality, hypertension, diabetes, hyperlipemia, take ototoxic drug, occupational noise exposure (not in ARHL/NIHL subgroup analyses), stroke.

**Table S7.** Sensitive analyses on associations of PRS calculated by 8 SNPs with hearing loss ^a^**.**

| Population | | PRS | | | | |
| --- | --- | --- | --- | --- | --- | --- |
|  |  | Continuous ^b^ | Low | Medium | High | *P*-trend |
| Overall (n=15827) |  | **1.54 (1.41, 1.69)** | 1.00 (ref.) | **1.13 (1.04, 1.23)** | **1.44 (1.32, 1.57)** | <0.001 |
| ARHL subgroup (n=9456) |  | **1.56 (1.39, 1.76)** | 1.00 (ref.) | **1.14 (1.02, 1.28)** | **1.47 (1.31, 1.64)** | <0.001 |
| NIHL subgroup (n=6170) |  | **1.51 (1.31, 1.74)** | 1.00 (ref.) | **1.12 (0.98, 1.28)** | **1.39 (1.21, 1.59)** | <0.001 |

Model adjusted for age, gender, body mass index, education level, smoking status, drinking status, regular exercise, sleep quality, hypertension, diabetes, hyperlipemia, take ototoxic drug and occupational noise exposure (not in ARHL/NIHL subgroup analyses).

^a^ 8 SNPs were selected from 37 SNPs with *P*< 0.01 in Table S1.

^b^ The OR (95%CI) of hearing loss when PRS increased 5 risk alleles.


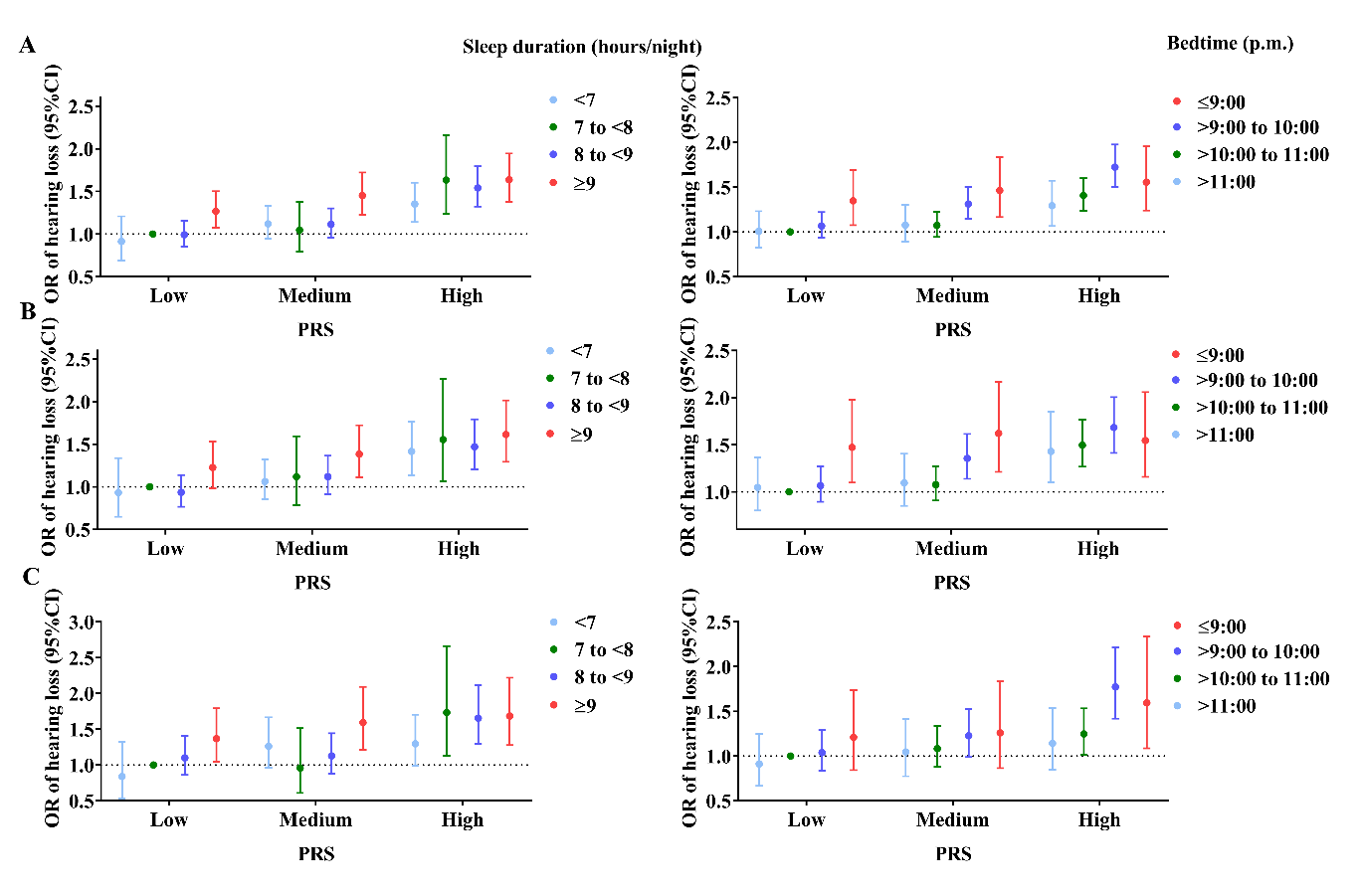


**Fig.S4.** Joint associations of sleep duration and PRS, or bedtime and PRS calculated by 8 SNPs with hearing loss in overall (A), ARHL subgroup (B) and NIHL subgroup (C). References were set at the low level of PRS and sleep duration of 7 to <8 hours/night, or the low level of PRS and bedtime of >10:00 p.m. to 11:00 p.m..

**Table S8. Interactions between sleep duration and PRS** **calculated by 8 SNPs on the risk of hearing loss among those with bedtime ≤10:00 p.m.**

| Sleep duration (hours/night) | | PRS | | | | | | *P*-_int_ |
| --- | --- | --- | --- | --- | --- | --- | --- | --- |
|  |  | Low | | Medium | | High | |  |
|  |  | Case/Total | OR (95%CI) | Case/Total | OR (95%CI) | Case/Total | OR (95%CI) |  |
| Overall (n=6934) | |  |  |  |  |  |  | **0.018** |
|  | 7 to <8 | 115/197 | 1.00 (ref.) | 116/206 | 1.00 (ref.) | 108/199 | 1.00 (ref.) |  |
|  | 8 to <9 | 466/1025 | **0.66 (0.47, 0.92)** | 540/1030 | 0.96 (0.69, 1.33) | 604/1020 | **1.43 (1.03, 2.00)** |  |
|  | ≥9 | 589/1087 | 0.89 (0.63, 1.25) | 634/1090 | 1.23 (0.89, 1.71) | 668/1080 | **1.53 (1.10, 2.13)** |  |
| ARHL subgroup (n=4170) | |  |  |  |  |  |  | 0.018 |
|  | 7 to <8 | 66/111 | 1.00 (ref.) | 68/116 | 1.00 (ref.) | 66/124 | 1.00 (ref.) |  |
|  | 8 to <9 | 273/636 | **0.56 (0.36, 0.88)** | 315/622 | 0.70 (0.45, 1.09) | 355/626 | 1.46 (0.96, 2.23) |  |
|  | ≥9 | 338/632 | 0.81 (0.51, 1.27) | 367/654 | 0.95 (0.61, 1.48) | 388/650 | 1.49 (0.98, 2.27) |  |
| NIHL subgroup (n=2660) | |  |  |  |  |  |  | 0.557 |
|  | 7 to <8 | 46/83 | 1.00 (ref.) | 43/84 | 1.00 (ref.) | 41/72 | 1.00 (ref.) |  |
|  | 8 to <9 | 182/378 | 0.87 (0.52, 1.48) | 215/396 | 1.53 (0.92, 2.55) | 236/378 | 1.33 (0.75, 2.35) |  |
|  | ≥9 | 238/437 | 1.09 (0.64, 1.85) | 252/420 | **1.82 (1.09, 3.04)** | 264/412 | 1.51 (0.85, 2.66) |  |

Model adjusted for age, gender, body mass index, education level, smoking status, drinking status, regular exercise, sleep quality, hypertension, diabetes, hyperlipemia, take ototoxic drug and occupational noise exposure (not in ARHL/NIHL subgroup analyses).

**Table S9.** **Interactions between bedtime and PRS** **calculated by 8 SNPs on the risk of hearing loss among those with sleep duration ≥9 hours/night**

| Bedtime (p.m.) | | PRS | | | | | | *P*-_int_ |
| --- | --- | --- | --- | --- | --- | --- | --- | --- |
|  |  | Low | | Medium | | High | |  |
|  |  | Case/Total | OR (95%CI) | Case/Total | OR (95%CI) | Case/Total | OR (95%CI) |  |
| Overall (n=4038) | |  |  |  |  |  |  | 0.087 |
|  | ≤10:00 | 587/1085 | 0.93 (0.67, 1.27) | 628/1078 | 1.26 (0.92, 1.73) | 676/1094 | **1.59 (1.15, 2.19)** |  |
|  | >10:00 to 11:00 | 114/260 | 1.00 (ref.) | 104/269 | 1.00 (ref.) | 110/252 | 1.00 (ref.) |  |
| ARHL subgroup (n=2382) | |  |  |  |  |  |  |  |
|  | ≤10:00 | 338/632 | 0.97 (0.63, 1.48) | 362/645 | **1.71 (1.12, 2.60)** | 393/659 | 1.18 (0.78, 1.79) | 0.144 |
|  | >10:00 to 11:00 | 65/148 | 1.00 (ref.) | 49/155 | 1.00 (ref.) | 69/143 | 1.00 (ref.) |  |
| NIHL subgroup (n=1596) | |  |  |  |  |  |  |  |
|  | ≤10:00 | 236/435 | 0.90 (0.54, 1.49) | 253/419 | 0.80 (0.49, 1.33) | 265/415 | **2.87 (1.65, 5.00)** | **0.001** |
|  | >10:00 to 11:00 | 49/109 | 1.00 (ref.) | 54/113 | 1.00 (ref.) | 37/105 | 1.00 (ref.) |  |

Model adjusted for age, gender, body mass index, education level, smoking status, drinking status, regular exercise, sleep quality, hypertension, diabetes, hyperlipemia, take ototoxic drug and occupational noise exposure (not in ARHL/NIHL subgroup analyses).


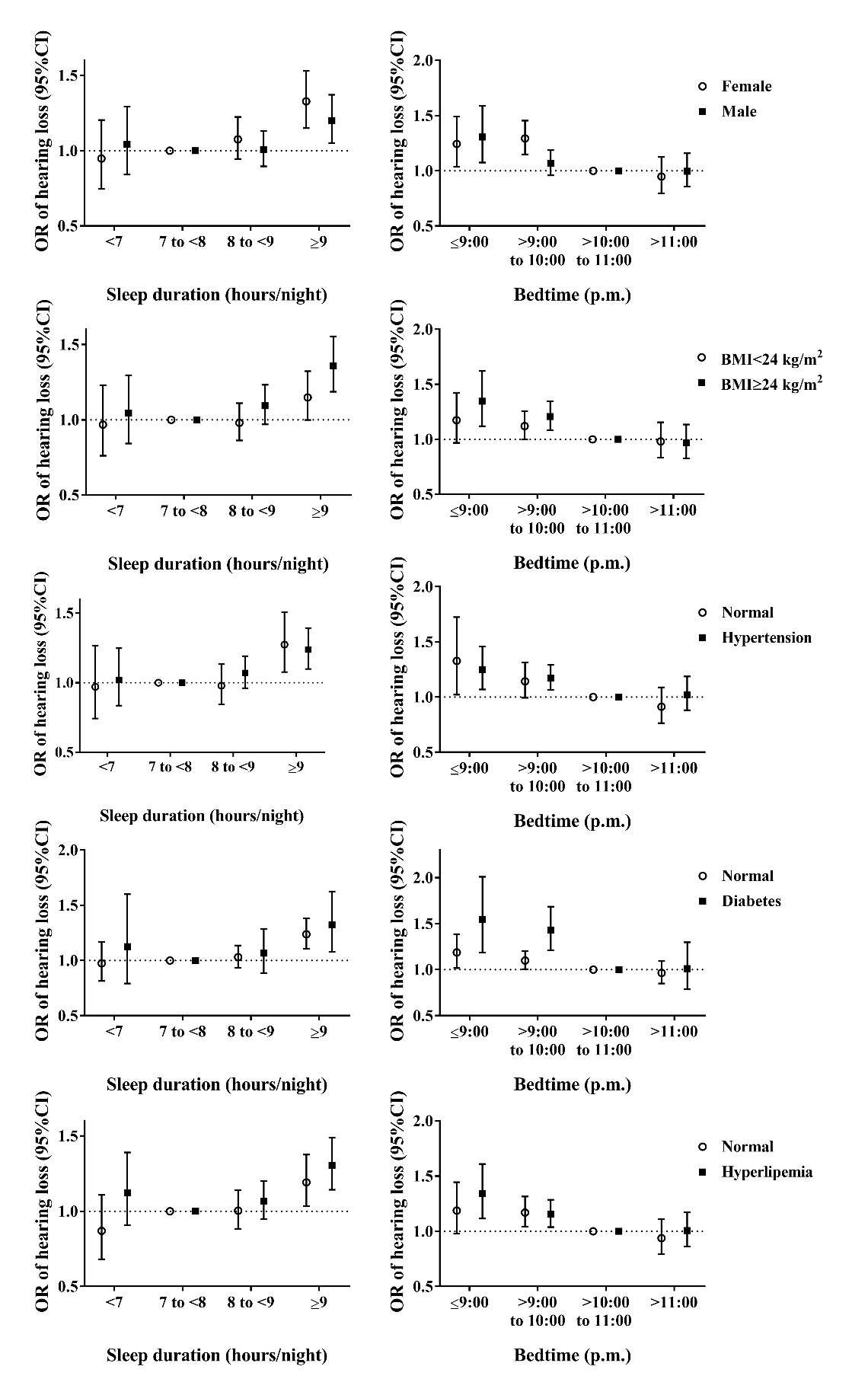


**Fig. S5.** Associations of sleep duration and bedtime with hearing loss stratified by major characteristics in overall.


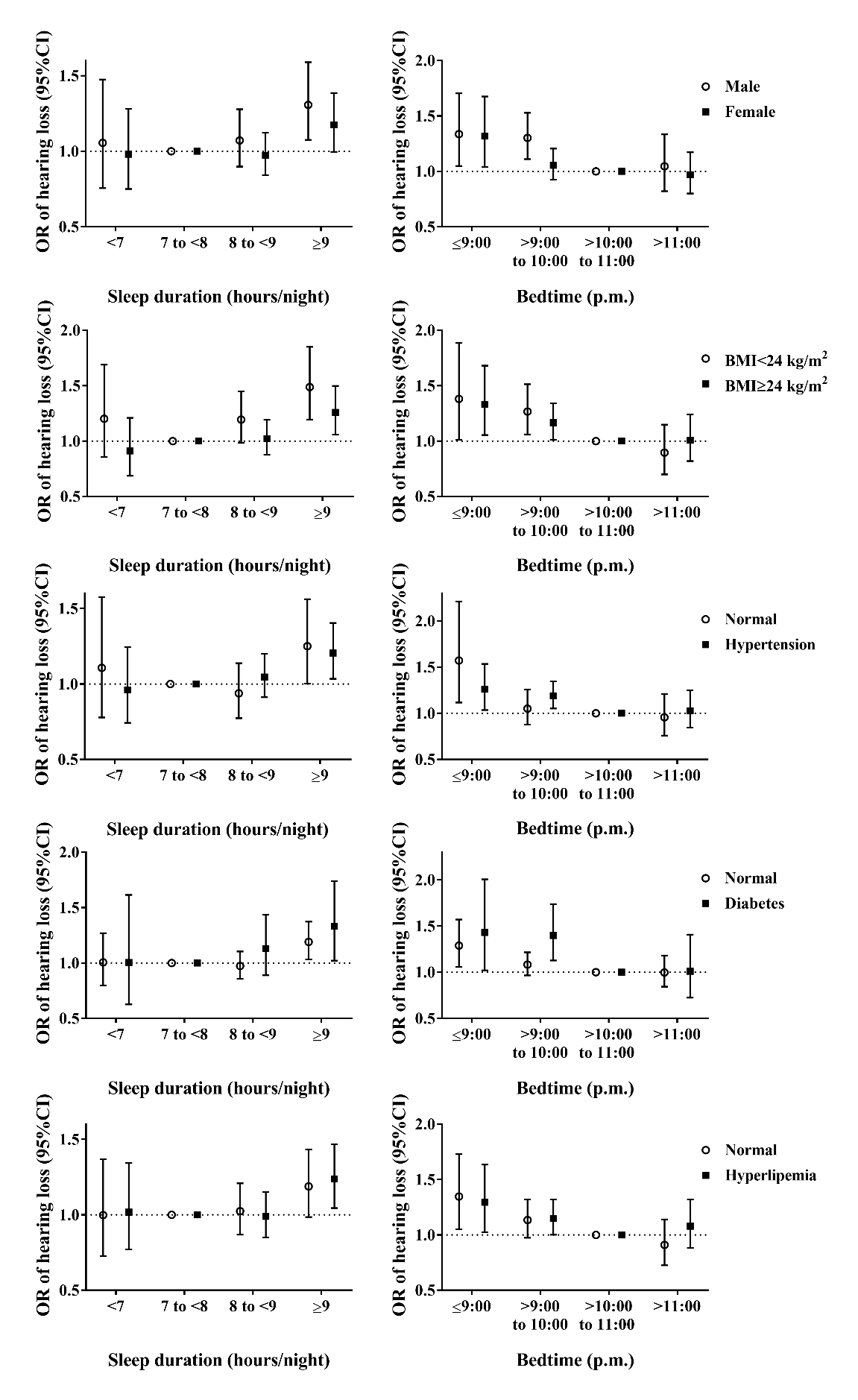


**Fig. S6.** Associations of sleep duration and bedtime with hearing loss stratified by major characteristics in ARHL subgroup.


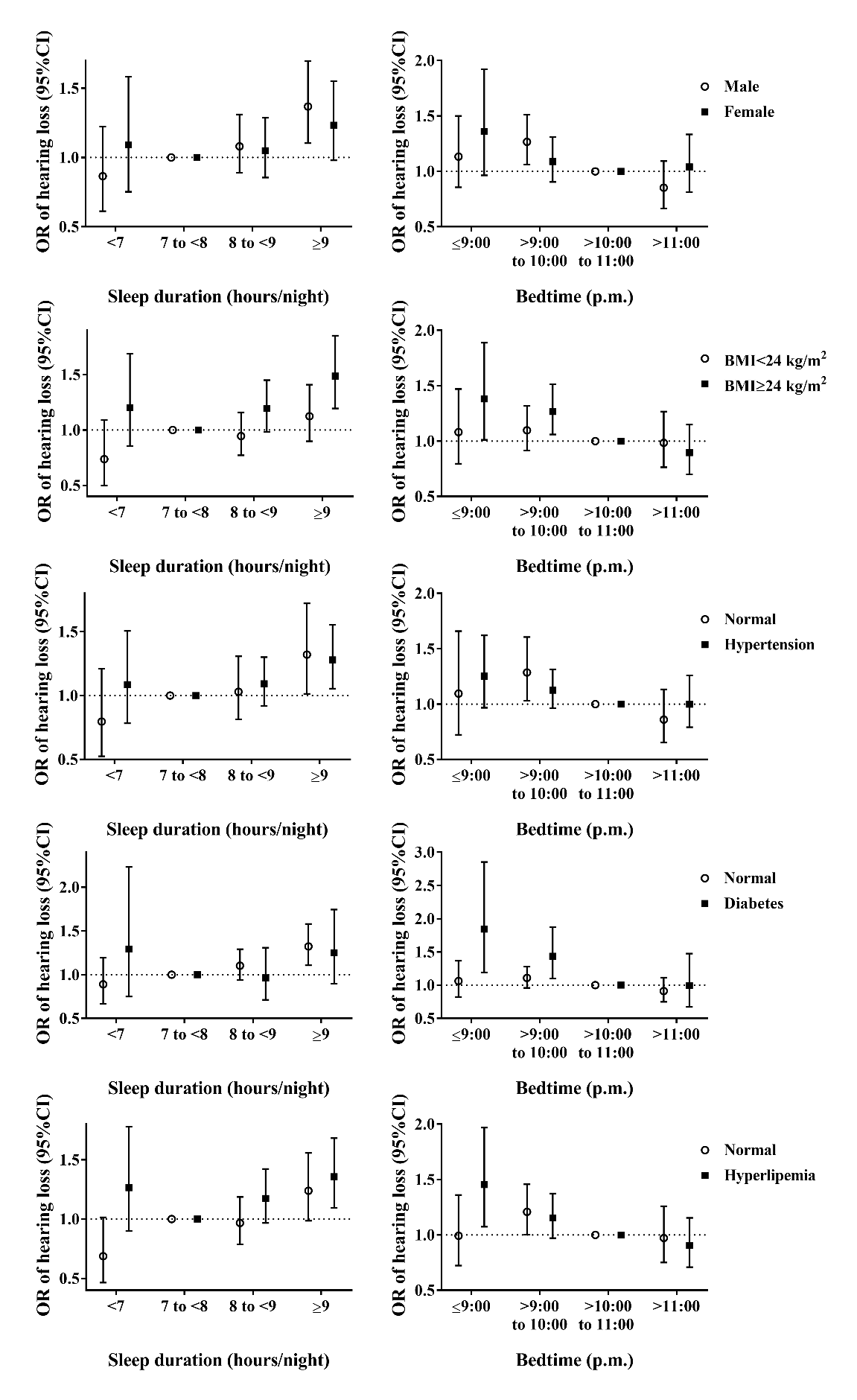


**Fig. S7.** Associations of sleep duration and bedtime with hearing loss stratified by major characteristics in NIHL subgroup.
